# Exploring the interplay of new energy vehicle enterprises, consumers, and government in the context of the "dual carbon" target: An evolutionary game and simulation analysis

**DOI:** 10.1371/journal.pone.0291175

**Published:** 2023-09-08

**Authors:** Rui Song, Wen Shi, Wanyi Qin, Xingjian Xue

**Affiliations:** 1 School of Logistics and Transportation, Central South University of Forestry and Technology, Changsha, China; 2 School of Landscape Architecture, Central South University of Forestry and Technology, Changsha, China; PLOS, UNITED KINGDOM

## Abstract

To achieve low-carbon and green mobility, the government needs to encourage people to buy and use new energy vehicles. This study proposes a tripartite evolutionary game model among new energy vehicle manufacturers, consumers, and government agencies. The game strategy combinations of each party and the stability conditions of the equilibrium point in the evolutionary game system are analyzed, and the validity of the conclusions is verified by simulation results. Compared with traditional studies that suggest the government should adopt the direct subsidy policy, this study shows that in the early stage of new energy vehicle development, government subsidies are still important for the rapid growth of new energy vehicle production and sales, but indirect policies can play a key role as the new energy vehicle industry matures. In addition to the price, the attractiveness of vehicle brands, the perceived utility of the products among consumers, and the coverage of charging infrastructure in cities also determine whether consumers decide to purchase and use new energy vehicles. The findings could provide useful recommendations for governments and manufacturers of new energy vehicles to meet their "dual carbon" targets.

## Introduction

Achieving the "carbon peak" and "carbon neutral" targets is a significant strategic decision made by the Chinese government after careful consideration. Green and low-carbon mobility has become a consensus among the whole society. In the government report of the National People’s Congress in 2021, China clearly proposed to achieve "peak carbon" by 2030 and "carbon neutral" by 2060 [1]. However, being a developing country, China’s energy consumption is dominated by traditional coal and oil. Although the growth rate of greenhouse gas emissions has slowed down in recent years, emissions from vehicles in the transport field are still on the rise. While people enjoy the fast, convenient, and comfortable travel that transport brings, carbon emissions from transport are also increasing year by year. Statistics show that China is currently one of the largest carbon-emitting countries in the world, and the transport sector ranks third in terms of carbon emissions, accounting for approximately 10% of all industries [2]. This indicates that China has a colossal task and challenge to successfully achieve the "dual carbon" target. To meet this goal, China needs to actively promote energy-efficient and low-carbon transport and encourage urban residents to switch to low-carbon and green travel as soon as possible.

New energy vehicles are powered by green and environmentally friendly electricity, in contrast to traditional fuel vehicles that rely on non-renewable oil resources. However, the adoption of new energy vehicles in China remains low. As of June 2021, the Ministry of Public Security reported that out of the 384 million vehicles in the country, only 6.03 million were new energy vehicles, accounting for just 2.06% of the total [2]. To promote the healthy growth of the new energy vehicle industry, China has long supported its development. In 2021, the State Council released the “Action Plan to Reach the Carbon Peak by 2030,” which set a target for new energy and clean energy-powered vehicles to reach approximately 40% of all vehicles on the road by 2030, with an estimated 10 million new energy vehicles purchased that year [3]. To compete with conventional fuel vehicles and meet these ambitious goals, both government support and consumer demand are necessary. The governments can encourage car manufacturers to accelerate production of new energy vehicles, while also incentivizing consumers to increase their purchase and use of such vehicles for transportation. As part of the national strategy to promote low-carbon mobility, exploring the evolutionary game among new energy vehicle manufacturers, consumers, and the governments is crucial to achieving the goals of "carbon peak" and "carbon neutral" in the transport sector as quickly as possible.

In the past decade, China has witnessed rapid growth in the new energy vehicle market, becoming the largest in the world. However, practical issues remain to be solved. There are still a number of important factors that influence the government’s management of the new energy vehicle market, such as the effectiveness of government subsidies for manufacturers [4] and the willingness of consumers to purchase and use new energy vehicles [5]. These factors will also ultimately affect the development of China’s green and circular economy and the achievement of its "dual carbon" goals. While local governments offer subsidies or incentives to manufacturers of new energy vehicles, deficiencies in such policies exist. Some manufacturers find ways to cheat the government subsidies by setting up intermediary leasing companies and selling vehicles directly to each other [6], causing irreversible losses to the subsidy system, offering no real policy protection to consumers, and placing a significant burden on government finances in the long run. If subsidies for the production and consumption of new energy vehicles are gradually reduced or removed in the future, this may affect car manufacturers’ strategies in producing new energy vehicles or discourage users from buying them. Therefore, it is necessary to examine whether subsidies can be combined with other indirect incentives to promote the growth of the new energy vehicle market. It is also important to study the willingness of consumers to buy and use new energy vehicles. What specific factors determine whether consumers buy new energy vehicles or conventional fuel vehicles, and whether they choose to travel by car or by public transport, are all matters that need to be explored in depth. Many consumers remain skeptical about purchasing or using new energy vehicles due to concerns about the stability of the development process and the lack of complete charging infrastructure. Consumers’ willingness to buy and use new energy vehicles as their primary mode of transportation depends largely on their evolutionary game strategy.

## Literature review

The existing literature mainly focuses on discussing the effectiveness of government policies on new energy vehicles. It also analyzes the strategic combination choice in the dynamic game relationship between two or three parties. Moreover, the literature explores the consumers’ willingness to purchase new energy vehicles. However, there is a need for further research in this field to gain a more comprehensive understanding of the topic.

Specifically, scholars have made a detailed analysis of various new energy vehicle policies adopted by the government, which were embodied in quota subsidies for the production of new energy vehicles by manufacturers [5, 7], direct subsidies for technological research and development of new energy vehicle manufacturers [8–10], and exemption or reduction of vehicle tax for new energy vehicles, etc. [11–13]. Scholars believed that direct subsidies were an effective policy to stimulate manufacturers to produce more new energy vehicles. Gong H, et al. [14], by analyzing the changing pattern of new energy vehicle policies in China over the past decade, concluded that direct government subsidies for the new energy vehicle production were a direct and effective policy. Zhong T Y, et al. [15] argued that direct subsidies and incentives should be increased at the early stage of the development of new energy vehicles in order to achieve the purpose of increasing the market share. Zhang L, et al. [16] constructed an evolutionary game model of the interaction of local government environmental regulation strategies in China from the perspective of direct government subsidies. In addition, some scholars believed that in addition to direct subsidies to new energy vehicle manufacturers, other indirect policies also affected the development of new energy vehicles. For example, Zhou Y, et al. [17] conducted a comparative analysis of the marginal utility of fiscal subsidies and tax rebates, and found compared to fiscal subsidies, tax breaks were more market-oriented. Zhang X L, et al. [18] argued that the governments did not need to choose a reasonable subsidy policy but can also adopted a regulatory penalty mechanism for conventional fuel vehicles. Zhang S Y, et al. [19] examined the impact of government indirect policies on corporate carbon emissions under two scenarios: a static carbon trading price and a dynamic carbon trading price.

It was also of great practical significance to study the purchase intention and influencing factors of new energy vehicles from the perspective of consumers, and it was also one of the important criteria to judge whether the government’s new energy vehicle policy could achieve the expected effect. Ma S H, et al. [20] adopted a preference choice experiment to examine the individual characteristics of consumers, vehicle attributes and other variables, and their findings showed that the middle class had a higher willingness to accept new energy vehicles. Xu G H, et al. [21] used questionnaires and principal component analysis to conduct factor analysis, and the results showed that urban residents had a relatively high level of awareness of new energy vehicles and also had a certain willingness to purchase them. Chen K, et al. [22] investigated consumers’ willingness to purchase new energy vehicles based on a perceived benefit and perceived risk framework and showed that environmental awareness had a significant positive effect on the willingness to purchase new energy vehicles. Helveston J P, et al. [23] analyzed the differences in consumer acceptance of new energy vehicles and traditional vehicles between China and the US based on consumer survey data from the two countries. Li X W, et al. [24] developed a game model for recycling units and consumers in two different scenarios with and without remanufacturing capacity.

Dynamic evolutionary game models are used to study the behavioral and strategic choices of competition and cooperation. Through the construction and analysis of evolutionary game models, it is possible to gain insight into the mechanisms of behavior and strategy choice in different domains and to predict the interactions and effects of different strategies. The application of evolutionary game models can provide guidance on decision-making and strategy choice for practical problems. Many scholars have mainly used the two-party evolutionary game model between the government, car manufacturers and consumers [25]. Ji S F, et al. [26] constructed a two-party game model between the behavior of automotive enterprises and government subsidies, and conducted an econometric analysis of various influencing factors. Based on the two-party evolutionary game model, Shi Y Y, et al. [27] studied the impact of firms’ strategic choices and consumers’ decisions on the diffusion of low-carbon technologies. Syed A R K, et al. [28] empirically investigated the impact of green capabilities and green procurement practices on the bottom line performance of manufacturing firms. Khan S A R, et al. [29] ’s findings showed that in the two-party evolutionary game, advanced logistics infrastructure and renewable energy consumption played a key role in increasing economic growth and international tourism. Dou Y D, et al. [30] constructed a two-party dynamic game evolution model of "formal group—informal group". Wang G, et al. [31] presented a public-private partnership project, using a two-party evolutionary game theory approach to assess the impact of typical factors on the project. In addition, a number of other dynamic analysis models were widely used. Zhou C Y, et al. [32] used the Stackelberg game approach to reveal the mechanisms of innovative behavior of recyclers in a CDW recycling PPP project. Jiang N, et al. [33] developed a disjunctive game model for the evolution of green and low carbon (GLC) innovation in manufacturing firms.

The research objective of this paper is to study the game strategies adopted by various stakeholders, including new energy vehicle manufacturers, consumers and government agencies, and the equilibrium conditions of the evolutionary game system in the context of "dual carbon". By constructing a three-party evolutionary game model, this paper proposes relevant measures and recommendations based on the simulation results and proves the validity of the research findings.

Compared to other research findings, this study is distinguished as follows:(1) Traditional research has suggested that governments can adopt either direct subsidy policies or indirect policies to increase the incentive for manufacturers to produce new energy vehicles [34]. However, the question of whether a combination of direct and indirect policies can be used, and at what stage, remains a pressing one. This paper confirms a multi-faceted approach combining direct subsidies and indirect support strategies is needed for the government to promote the rapid development of the new energy vehicle industry. As the industry matures, indirect policies will play a key role in regulating the new energy vehicle market. (2) According to the literature cited in this paper [5], manufacturers must reduce the price of new energy vehicles to encourage consumer purchase. The findings of this paper suggest that in a three-way evolutionary game system, it is more important for car manufacturers to have differentiated core technologies to increase the attractiveness of their brands and improve the perceived utility of their products among consumers, and for government departments to increase the coverage of charging infrastructure in their cities to increase consumer interest in using new energy vehicles.(3)Through numerical simulations, this paper provides a more intuitive understanding of the game process and sensitivity analysis of key factors, providing policy insights for China to achieve sustainable green and healthy development.

In the remainder of this paper, we first explain the model assumptions and framework, and then delve into an analysis of the game strategies employed by car manufacturers, consumers, and governments. Then, we provide simulation analysis and subsequent discussion. Finally, we summarize our findings and provide recommendations for future research.

## Model assumptions and framework construction

**Fig 1:**
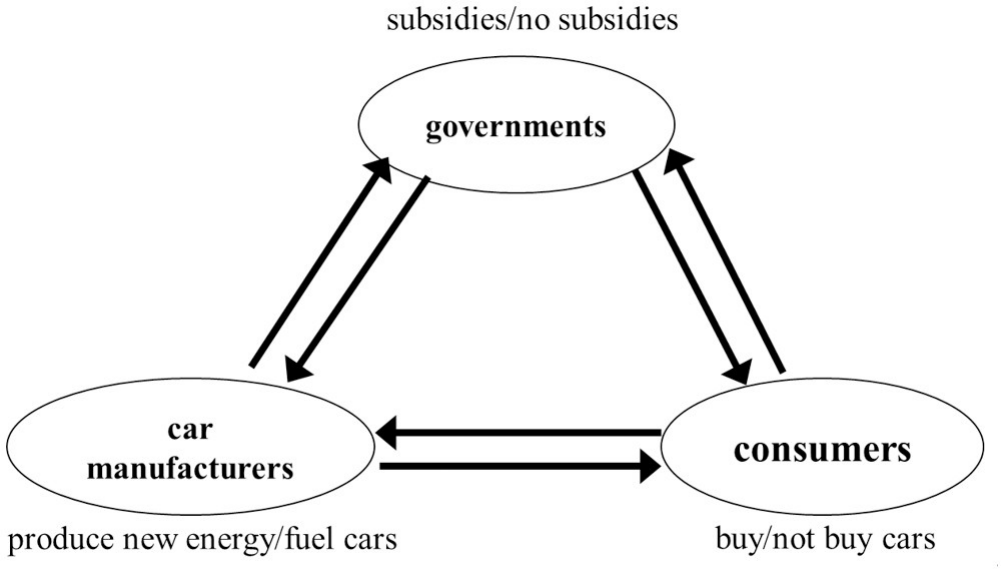
Framework of the relationship between the parties in the three-party evolutionary game system.

In order to study the game relationship among car manufacturers, consumers and the governments, the framework of the tripartite evolutionary game model is shown in Fig 1. It depicts the relationship between new energy vehicle manufacturers, consumers, and governments in a tripartite dynamic evolutionary system. The manufacturer can choose to produce a new energy vehicle or a conventional fuel vehicle, the consumer can choose to buy the vehicle or not, and the government can choose to fund the new energy vehicle or not. Several assumptions need to be made as follows:

Hypothesis 1: The players in the three-party evolutionary game model include car manufacturers, consumers, and government departments. It is assumed that all three participants are finite rational agents, and the game strategy of each party will evolve over time and eventually stabilize at the optimal strategy [23].

Hypothesis 2: The strategy space for car producers is to produce new energy cars or traditional fuel ones. The probability of car producers choosing to produce new energy cars is *x*, and the probability of choosing to produce fuel cars is 1 − *x*, where *x* ∈ [0,1]; the strategy space for consumers is to buy cars or not. The probability of consumers to buy cars is *y*, the probability of not to buy cars is 1 − *y*, where *y* ∈ [0,1]; the strategy space for government departments is to make subsidies or not. The probability of government departments to make subsidies for the production or consumption of new energy cars is *z*, the probability of not making subsidies is 1 − *z*, where *z* ∈ [0,1] [35].

Hypothesis 3: For car manufacturers, they can choose to produce either new energy vehicles or conventional fuel ones, and the sales revenue of the two types of vehicles are *π*_1_ and *π*_2_ respectively. If the car manufacturers choose to produce new energy vehicles, the government subsidies received are *R*_2_, and the additional earnings received through carbon trading management are *R*_3_. If the car manufacturers choose to produce conventional fuel vehicles, the fines of governments are *F* [36].

Hypothesis 4: Consumers can choose to buy cars, not to buy and choose other public transport. Assume that the utility values of new energy vehicles and fuel vehicles for consumers are *V*_1_ and *V*_2_ respectively, where *V*_1_ > *V*_2_ [37]. The difference between the two values includes the additional government subsidy for purchasing new energy vehicles and the importance of green living for consumers; the prices of new energy vehicles and fuel vehicles are *P*_1_ and *P*_2_ respectively; *n*_1_ is the coverage of public charging posts in consumers’ cities, *η*_1_ is the sensitivity of consumers to the coverage of charging posts; Λ is the vehicle purchase tax. In addition, *C*_1_ and *C*_2_ denote the cost per unit distance travelled for new energy vehicles and fuel ones respectively, and *L* is the vehicle mileage travelled. If consumers do not purchase a car and chose public transport, the total travel cost spent is *C*_6_, and the benefit of the government subsidy for low-carbon travel is *R*_4_.

Hypothesis 5: If the governments adopt subsidy policies, the human resource cost to be spent is *C*_3_, and the environmental benefits that the governments can obtain are *R*_1_ [23]. If the governments do not adopt subsidy policies for new energy vehicles, the time cost of society’s gradual shifting from high-carbon and high-pollution travels to low-carbon and green travels is *C*_5_, and the environmental governance cost to the governments is *C*_4_.

Based on the above assumptions, the three-way evolutionary game matrix between car manufacturers, consumers and governments are shown in Table 1. Table 1 shows the game matrix between the producers of new energy vehicles, consumers, and governments. The matrix measures the revenue or loss from different combinations of game strategies chosen by each party in the evolutionary game system.

**Table 1:** Game matrix for the evolutionary combination strategy of car manufacturers, consumers and governments.

Consumers			Governments	
			subsidies z	no subsidies 1-z
**Vehicle Manufacturers**	produce new energy vehicles x	purchase y	$\begin{array}{l} \pi_{1}\text{+}R_{2}+R_{3};\\ V_{1}-P_{1}+n_{1}\eta_{1}-C_{1}L;\\ R_{1}-C_{3}-R_{2} \end{array}$	$\begin{array}{l} \pi_{1}+R_{3};\\ V_{1}-P_{1}+n_{1}\eta_{1}-C_{1}L;\\ R_{1}-C_{5} \end{array}$
		not purchase 1-y	$\begin{array}{l} R_{2}+R_{3};\\ R_{6}-C_{6};\\ R_{1}-C_{3}-R_{2} \end{array}$	$\begin{array}{l} R_{3};\\ -C_{6};\\ R_{1}-C_{5} \end{array}$
	produce fuel cars 1-x	purchase y	$\begin{array}{l} \pi_{2}-F;\\ V_{2}-P_{2}-\Lambda -C_{2}L;\\ F-C_{3}-C_{4} \end{array}$	$\begin{array}{l} \pi_{2}-F;\\ V_{2}-P_{2}-\Lambda -C_{2}L;\\ F-C_{5}-C_{4} \end{array}$
		not purchase 1-y	$\begin{array}{l} -F;\\ R_{6}-C_{6};\\ F-C_{3} \end{array}$	$\begin{array}{l} -F;\\ -C_{6};\\ F-C_{5} \end{array}$

## Model analysis

### Analysis of the game strategies of car manufacturers

Let *E*_11_ be the expected return to the car manufacturer from producing new energy vehicles, *E*_12_ be the return from producing fuel vehicles and $\bar{E_{1}}$ be the average expected return, then the following relationship exists:

$$\left\{\begin{array}{l} E_{11}=yz\left[\pi_{1}+R_{2}+R_{3}\right]+y(1-z)[\pi_{1}+R_{3}]+(1-y)z[R_{2}+R_{3}]+(1-y)(1-z)R_{3}\\ E_{12}=yz\left[\pi_{2}-F\right]+y(1-z)[\pi_{2}-F]+(1-y)z[-F]+(1-y)(1-z)[-F]\\ \bar{E_{1}}=xE_{11}+(1-x)E_{12} \end{array}\right.$$ (1)

Further simplification of Eq (1) yields:

$$\left\{\begin{array}{l} E_{11}=yz\pi_{1}+zR_{2}+R_{3}\\ E_{12}=y\pi_{2}-F\\ \bar{E_{1}}=xE_{11}+(1-x)E_{12}\text{=}xyz\pi_{1}+xzR_{2}+xR_{3}+y\pi_{2}-F-xy\pi_{2}+xF \end{array}\right.$$ (2)

Using Eq (2), the replication dynamic equation for the car manufacturers’ choice of strategy is:

$$F(x)=\frac{dx}{dt}=x(E_{11}-\bar{E_{1}})=x(1-x)[yz\pi_{1}+zR_{2}+R_{3}-y\pi_{2}+F]$$ (3)

Through Eq (3), the first derivative of *F*(*x*) with respect to *x* can be calculated as:

$$\frac{d(F(x))}{dx}=(1-2x)[yz\pi_{1}+zR_{2}+R_{3}-y\pi_{2}+F]$$ (4)

**Fig 2:**
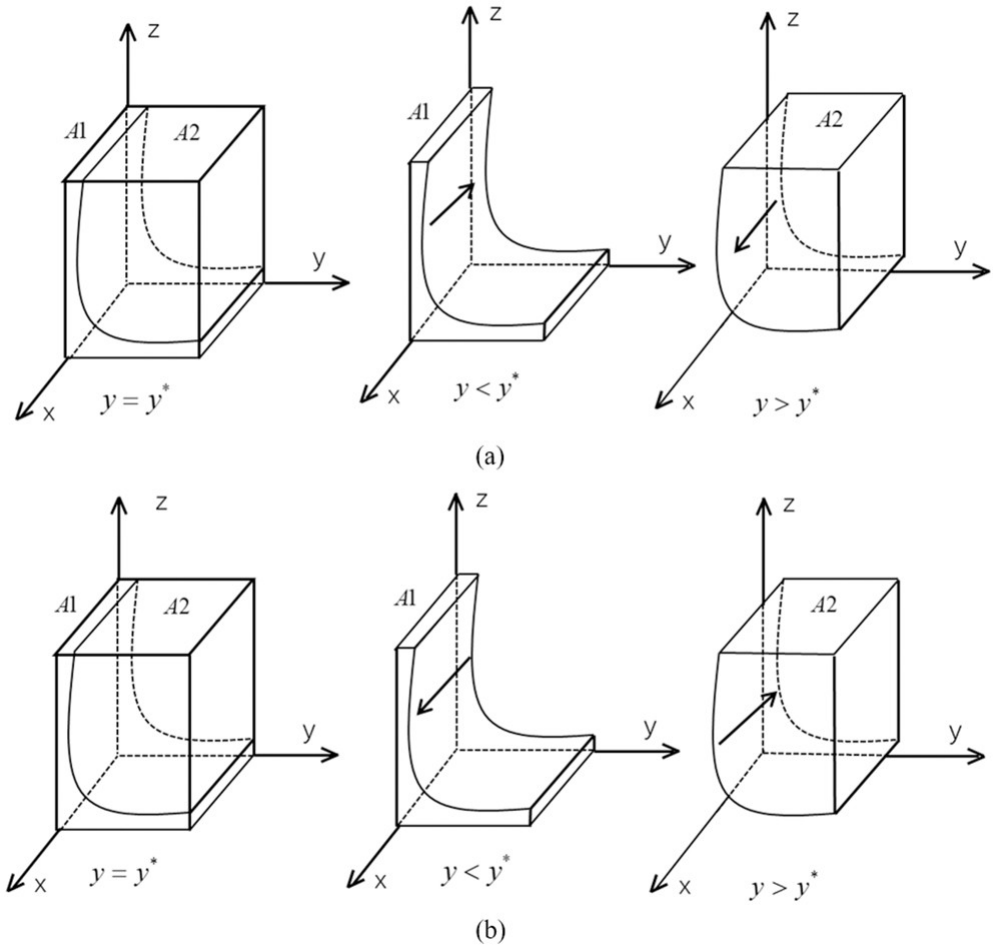
The evolution phase diagram of car manufacturers.

According to the stability condition theorem for differential equations, the following conditions must be satisfied for the probability of car manufacturers producing new energy vehicles to reach a steady state: *F*(*x*) = 0 and *d*(*F*(*x*))/*dx* < 0. If *G*(*y*) = *yzπ*_1_ + *zR*_2_ + *R*_3_ − *yπ*_2_ + *F* and *z* > *π*_2_/*π*_1_, then *dG*(*y*)/*dy* > 0, and *G*(*y*) is increasing functions with respect to *y*; if *z* < *π*_2_/*π*_1_, then *dG*(*y*)/*dy* < 0, and *G*(*y*) is decreasing functions with respect to *y*. Therefore, the following conclusions can be drawn: when *y* = y* = (2*R*_2_ + *R*_3_ + *F*)/(*π*_2_ − *zπ*_1_), *d*(*F*(*x*))/*dx* = 0 and *F*(*x*) = 0, *x* is in an evolutionary stable state. When *z* > *π*_2_/*π*_1_ and *y* > y*, *G*(*y*) > 0, it can be concluded that *d*(*F*(*x*))/*dx*|_*x* = 1_ < 0 and *F*(*x*)|_*x* = 1_ = 0, then *x* = 1 is an evolutionary stable point for the car producer; conversely, when *y* < y*, then *x* = 0 is an evolutionary stable point. Similarly, if *z* < *π*_2_/*π*_1_, when *y* > y*, *x* = 0 is an evolutionary stable point; conversely, *x* = 1 is an evolutionary stable point. The evolutionary phase diagram of the auto producer’s policy is shown in Fig 2.

(a) *z* > *π*_2_/*π*_1_
(b) *z* < *π*_2_/*π*_1_

From Fig 2, it can be concluded that when *z* > *π*_2_/*π*_1_, the probability of the car producer maintaining stable production of fuel cars is the volume *V*_*A*1_, and the probability of stable production of new energy cars is the volume *V*_*A*2_. When *z* < *π*_2_/*π*_1_, the probability of the car producer maintaining stable production of new energy cars is the volume *V*_*A*1_, and the probability of stable production of fuel cars is the volume *V*_*A*2_. The calculations of *V*_*A*1_ and *V*_*A*2_ are shown below:

$$\begin{array}{l} V_{A1}=\int_{0}^{1}\int_{0}^{1}\frac{2R_{2}+R_{3}+F}{\pi_{2}-z\pi_{1}}dxdz=\frac{2R_{2}+R_{3}+F}{\pi_{1}}\text{ln}\frac{\pi_{2}}{\pi_{2}-\pi_{1}}\\ V_{A2}=1-V_{A1} \end{array}$$ (5)

Corollary 1: In the early stages of new energy vehicle development, there is an evolutionary process whereby the probability of a car manufacturer producing a new energy vehicle increases with the probability of government new energy subsidies and the probability of consumers purchasing a vehicle.

Proof: According to the game strategy analysis of auto producers, when *z* > *π*_2_/*π*_1_ and *y* > y*, *x* = 1 is the evolutionary stabilization point of auto producers, therefore, with the gradual increase of *y* and *z*, the stabilization strategy of auto producers gradually tends to *x* = 1 (i.e. producing new energy vehicles).

Corollary 2: If the difference in sales revenue between the production of new energy vehicles and conventional fuel vehicles is not significant, the probability of a car manufacturer producing new energy vehicles will be proportional to the government subsidies received, the additional revenue received through carbon trading management, and the government fines imposed on the car manufacturer for producing conventional fuel vehicles.

Proof: When *π*_1_ = *π*_2_ and *z* < 1, the first-order partial derivatives of *R*_2_, *R*_3_ and *F* on the *V*_*A*1_ is *∂V*_*A*1_/*∂R*_2_ > 0, *∂V*_*A*1_/*∂R*_3_ > 0, *∂V*_*A*1_/*∂F* > 0. Thus, increases in the values of *R*_2_, *R*_3_ and *F* all lead to an increase in the probability of an automobile manufacturer producing new energy vehicles.

Corollary 1 suggests that there is a strong correlation between government subsidies, production, and sales of new energy vehicles. In the early stages of the development of new energy vehicles, government subsidies as an evolutionary stabilization strategy are still important for the rapid growth of production and sales of new energy vehicles, as they are not yet fully competitive with fuel vehicles. If the governments reduce subsidies or removes subsidies, the price of new energy vehicles will increase significantly. Considering that many buyers of new energy vehicles are from the middle working class, they are very sensitive to price changes, so a significant price increase will inevitably affect their motivation to buy new energy vehicles, leading to a decline in sales and even a reduction in the production of new energy vehicles. On the other hand, if the sales of new energy vehicles continue to grow, it will directly stimulate the production of new energy vehicles and promote the development of the entire new energy vehicle industry.

Corollary 2 suggests that when the sales revenue of new energy vehicles and fuel vehicles are comparable, government departments can adopt direct policies such as increasing subsidies for new energy vehicles, as well as other indirect policies, such as increasing the additional revenues gained by new energy vehicle enterprises through carbon trading management and imposing penalties and restrictions on the production of fuel vehicles, all of which can serve the purpose of indirectly stimulating the growth of new energy vehicle production.

### Analysis of the gaming strategies of car consumers

Let *E*_21_ be the expected return of car consumers purchasing cars, *E*_22_ be the expected return of not purchasing cars, and $\bar{E_{2}}$ be the average expected return, then the following relationship exists:

$$\left\{\begin{array}{l} E_{21}=xz[V_{1}-P_{1}+n_{1}\eta_{1}-C_{1}L]+x(1-z)[V_{1}-P_{1}+n_{1}\eta_{1}-C_{1}L]+(1-x)z[V_{2}-P_{2}-\Lambda -C_{2}L]\\ +(1-x)(1-z)[V_{2}-P_{2}-\Lambda -C_{2}L]\\ E_{22}=xz[R_{4}-C_{6}]+x(1-z)[-C_{6}]+(1-x)z[R_{4}-C_{6}]+(1-x)(1-z)[-C_{6}]\\ \bar{E_{2}}=yE_{21}+(1-y)E_{22} \end{array}\right.$$ (6)

A further simplification of Eq (6) can be:

$$\left\{\begin{array}{l} E_{21}=x[V_{1}-P_{1}+\theta e_{1}+n_{1}\eta_{1}-C_{1}L]+(1-x)[V_{2}-P_{2}-\Lambda -C_{2}L]\\ E_{22}=-zR_{4}-C_{6}\\ \bar{E_{2}}=yE_{21}+(1-y)E_{22}=xy[V_{1}-P_{1}]+(1-x)y[V_{2}-P_{2}]\text{+}1-y[zR_{4}-C_{6}] \end{array}\right.$$ (7)

Combining Eq (7), the replication dynamics equation for car consumers is:

$$\begin{array}{l} F(y)=\frac{dy}{dt}=y(E_{21}-\bar{E_{2}})=y(1-y)[x(V_{1}-P_{1}+n_{1}\eta_{1}-C_{1}L)+(1-x)(V_{2}-P_{2}-\Lambda -C_{2}L)\\ -zR_{4}+C_{6}] \end{array}$$ (8)

The first order derivative of *F*(*y*) with respect to *y* is:

$$\begin{array}{l} \frac{d(F(y))}{dy}=(1-2y)[x(V_{1}-V_{2}-P_{1}+P_{2}+n_{1}\eta_{1}-\Lambda -C_{1}L+C_{2}L)+(V_{2}-P_{2}-\Lambda -C_{2}L)\\ -zR_{4}+C_{6}] \end{array}$$ (9)

**Fig 3:**
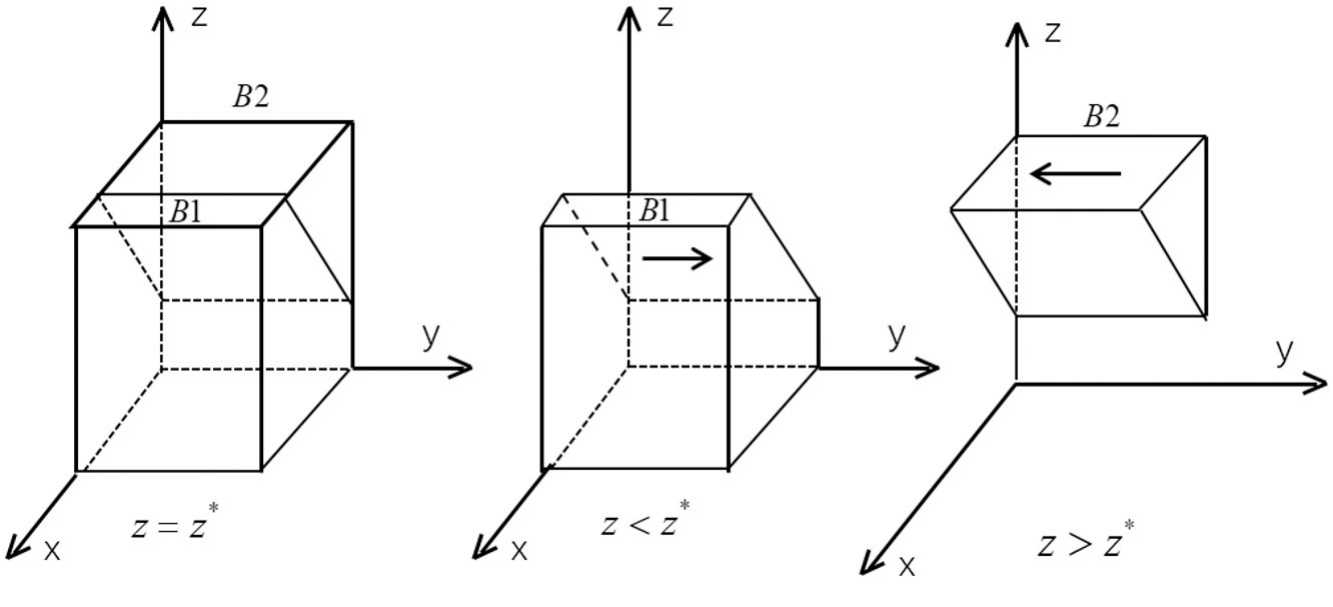
The evolution phase diagram of consumers.

According to the stability condition theorem of the differential equation, the probability of car consumers to buy new energy vehicles to reach a stable state must satisfy the following conditions: *F*(*y*) = 0 and *d*(*F*(*y*))/*dy* < 0. If we get *J*(*z*) = *x*(*V*_1_ ‒ *V*_2_ ‒ *P*_1_ + *P*_2_ + *n*_1_*η*_1_ ‒ Λ ‒ *C*_1_*L*+*C*_2_*L*) + (*V*_2_ ‒ *P*_2_ ‒ Λ ‒ *C*_2_*L*) ‒ *zR*_4_ +*C*_6_, and *J*(*z*) is a decreasing function with *z*, we can get the following conclusions: when *z* = *z* * = (*x*(*V*_1_ ‒ *V*_2_ ‒ *P*_1_ + *P*_2_ + *n*_1_*η*_1_ ‒ Λ ‒ *c*_1_*L*+*c*_2_*L*) + (*V*_2_ ‒ *P*_2_ ‒ Λ ‒ *c*_2_*L*) + *c*_3_*L*) / *R*_4_, *d*(*F*(*y*))/*dy* = 0, and *F*(*y*) = 0, then *y* is in the evolutionary game stable state. When *z* < z*, *J*(*z*) < 0, it can be concluded that *d*(*F*(*y*))/*dy*|_*y*=0_ < 0 and *F*(*y*)|_*y*=0_ = 0, then *y* = 0 is the consumers’ evolutionary stable point; similarly, when *z* < z*, *y* = 1 is the evolutionary stable point. The evolutionary phase diagram of the consumers’ strategy is shown in Fig 3.

From Fig 3, the probability of car consumers maintaining a steady purchase of cars is the volume *V*_*B*1_ and the probability of not purchasing cars is the volume *V*_*B*2_. The calculations of *V*_*B*1_ and *V*_*B*2_ are shown below.


$$\begin{array}{l} V_{B1}=\int_{0}^{1}\int_{0}^{1}\frac{x(V_{1}-V_{2}-P_{1}+P_{2}+n_{1}\eta_{1}-\Lambda -C_{1}L+C_{2}L)+V_{2}-P_{2}-\Lambda -C_{2}L+C_{6}}{R_{4}}dxd\text{y}\\ =\frac{V_{1}+V_{2}-P_{1}-P_{2}+n_{1}\eta_{1}-3\Lambda -C_{1}L-C_{2}L+2C_{6}}{2R_{4}}\\ V_{B2}=1-V_{B1} \end{array}$$ (10)

Corollary 3: The probability of purchasing new energy vehicles is positively related to the utility value of the new energy vehicle to the consumer and the coverage of public charging posts in the consumer’s city, and inversely related to the sales price of the new energy vehicle.

Proof: Given the first order partial derivatives of the probability *V*_*B*1_ with respect to *V*_1_, *n*_1_ and *P*_1_, we can derive *∂V*_*B*1_/*∂V*_1_ > 0, *∂V*_*B*1_/*∂n*_1_ > 0, and *∂V*_*B*1_/*∂P*_1_ < 0. Therefore, an increase in the values of *V*_1_ and *n*_1_ will lead to an increase in the probability of consumers buying new energy vehicles, and an increase in the value of *P*_1_ will lead to a decrease in the probability of consumers buying ones.

Corollary 3 suggests that consumers are sensitive to the price of new energy vehicles, and government departments and manufacturers should reduce the price of new energy vehicles if they want to increase the sales of new energy vehicles. In addition, government departments and manufacturers can take some other measures to increase the utility value of new energy vehicles to consumers, such as government efforts to promote low-carbon green mobility and manufacturers’ efforts to raise awareness of their products. In addition, by expanding the coverage of existing public charging piles in cities, the governments will also increase the consumption of new energy vehicles to a certain extent.

### Analysis of the gaming strategy of governments

Let *E*_31_ be the expected benefit of government departments subsidizing new energy vehicles, *E*_32_ be the expected benefit of no subsidy and $\bar{E_{3}}$ be the average expected benefit, then the following relationship exists:

$$\left\{\begin{array}{l} E_{31}=xy\left[R_{1}-C_{3}-R_{2}\right]+x(1-y)[R_{1}-C_{3}-R_{2}]+(1-x)y[F-C_{3}-C_{4}]+(1-x)(1-y)[F-C_{3}]\\ E_{32}=xy\left[R_{1}-C_{5}\right]+x(1-y)[R_{1}-C_{5}]+(1-x)y[F-C_{5}-C_{4}]+(1-x)(1-y)[F-C_{5}]\\ \bar{E_{3}}=zE_{31}+(1-z)E_{32} \end{array}\right.$$ (11)

Further simplification yields:

$$\left\{\begin{array}{l} E_{31}=x(R_{1}-C_{3}-R_{2})+(1-x)(F-C_{3})+(1-x)y(-C_{4})\\ E_{32}=x(R_{1}-C_{5})+(1-x)(F-C_{5})+(1-x)y(-C_{4})\\ \bar{E_{3}}=zE_{31}+(1-z)E_{32}=xR_{1}-zC_{3}-xzR_{2}+(1-x)F-(1-x)yC_{4}-(1-z)C_{5} \end{array}\right.$$ (12)

The replication dynamics equation for the governments is:

$$F(z)=\frac{dz}{dt}=z(E_{31}-\bar{E_{3}})=z(z-1)(xR_{2}+C_{3}-C_{5})$$ (13)

The first order derivative of *F*(*z*) with respect to *z* is:

$$\frac{d(F(z))}{dz}=(2z-1)(xR_{2}+C_{3}-C_{5})$$ (14)

**Fig 4:**
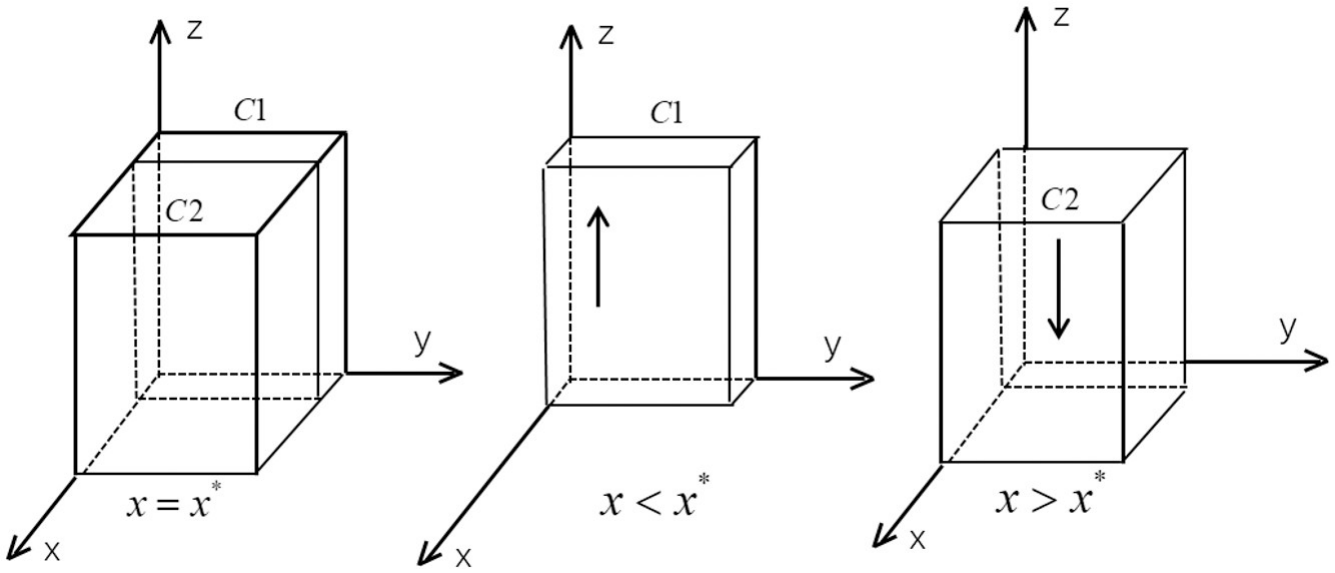
The evolution phase diagram of governments.

According to the stability condition theorem of the differential equation, the probability of the government department to subsidize new energy vehicles to reach a stable state must satisfy the following conditions: *F*(*z*) = 0 and *d*(*F*(*z*)) / *dz* < 0. When *x* = *x* * = (*C*_5_ ‒ *C*_3_) / *R*_2_, *d*(*F*(*z*)) / *dz* = 0, and *F*(*z*) = 0, all *z* are in the stable state of the evolutionary game. When *x* > *x* *, it follows that *d*(*F*(*z*)) / *dz* ⎹_*z*=0_ < 0 and *F*(*z*) ⎹_*z*=0_ = 0, *z* = 0 is the evolutionary stable point for the government sectors; conversely, when *x* < *x* *, *z* = 1 is the evolutionary stable point. The evolutionary phase diagram of the government departments’ strategy is shown in Fig 4.

From Fig 4, the probability of government subsidizing a new energy vehicle is the volume *V*_*c*1_, and the probability of no subsidy is the volume *V*_*c*2_. The calculations of *V*_*c*1_ and *V*_*c*2_ are shown below.


$$\begin{array}{l} V_{C1}=1*1*\frac{C_{5}-C_{3}}{R_{2}}=\frac{C_{5}-C_{3}}{R_{2}}\\ V_{C2}=1-V_{C1} \end{array}$$ (15)

Corollary 4: The probability of government subsidies for new energy policies is positively proportional to the time cost of changing society from high-carbon and high-pollution travel to low-carbon and green travel, and inversely proportional to the human resource cost of adopting government subsidies and the subsidy expenditure received to produce new energy vehicles.

Proof: Given the first-order partial derivative of the probability *V*_*c*1_ with respect to *C*_5_, *C*_3_ and *R*_2_, we can derive *∂V*_*C*1_ / *∂C*_5_ > 0, *∂V*_*C*1_ / *∂C*_3_ < 0, *∂V*_*C*1_ / *∂R*_2_ < 0. Thus, an increase in the value of *C*_5_ increases the probability of government subsidies for new energy vehicles, conversely, an increase in the values of *C*_3_ and *R*_2_ decreases the probability of subsidies.

Corollary 4 suggests that the probability of government subsidies for new energy policies is influenced by several factors. On the one hand, government subsidies are influenced by the time cost of changing from high-carbon, high-pollution travel to low-carbon, green travel. If the time cost is so high that the governments fail to meet the ’Double Carbon’ target, then government departments are likely to increase the probability of subsidizing new energy vehicles to avoid the penalties of public scrutiny and criticism. On the other hand, if the human cost of the government’s subsidy policy is too high or if the amount of subsidies for new energy vehicles continues to grow exponentially, this will have a further negative impact on the government’s finances.

### Stability analysis of equilibrium points in evolving systems

To simplify the calculation process, let *U*_1_ = *V*_1_ ‒ *P*_1_ + *n*_1_*η*_1_ ‒ *C*_1_*L*, *U*_2_ = *V*_2_ ‒ *P*_2_ ‒ Λ‒ *C*_2_*L*. Since *F*(*x*) = 0, *F*(*y*) = 0, *F*(*z*) = 0, and considering *x*, *y*, *z* ∊[0,1], the stability points of the three-way evolutionary game system can be found as follows: *E*_1_(0,0,0), *E*_2_(1,0,0), *E*_3_(0,1,0), *E*_4_(0,0,1), *E*_5_(1,1,0), *E*_6_(1,0,1), *E*_7_(0,1,1), *E*_8_(1,1,1), *E*_9_((*C*_5_ ‒ *C*_3_) / *R*_2_,1,(*π*_2_ ‒ 2*R*_2_ ‒ *R*_3_ ‒ *F*) / *π*_1_), *E*_10_(‒*U*_2_ ‒ *C*_6_ / *U*_1_ ‒ *U*_2_, 2*R*_2_ + *R*_3_ + *F* / *π*_2_, 0), *E*_11_ (*R*_4_ ‒ *U*_2_ ‒ *C*_6_ / *U*_1_ ‒ *U*_2_, 2*R*_2_ + *R*_3_ + *F* / *π*_2_ ‒ *π*_1_,1).

The Jacobi matrix of the three-party evolutionary game system can be computed as follows [28, 29]:

$$\begin{array}{l} J=\left(\begin{array}{ccc} J_{1} & J_{2} & J_{3}\\ J_{4} & J_{5} & J_{6}\\ J_{7} & J_{8} & J_{9} \end{array}\right)=\left(\begin{array}{ccc} \partial F(x)/\partial x & \partial F(x)/\partial y & \partial F(x)/\partial z\\ \partial F(y)/\partial x & \partial F(y)/\partial y & \partial F(y)/\partial z\\ \partial F(z)/\partial x & \partial F(z)/\partial y & \partial F(z)/\partial z \end{array}\right)=\\ \left(\begin{array}{ccc} \left(1-2x)(yz\pi_{1}+zR_{2}+R_{3}-y\pi_{2}+F\right) & x(1-x)(z\pi_{1}-\pi_{2}) & x(1-x)(y\pi_{1}+R_{2})\\ y(1-y)(U_{1}-U_{2}) & \left(1-2y)[x(U_{1}-U_{2})+U_{2}-zR_{4}+c_{3}L\right] & -y(1-y)R_{4}\\ z(z-1)R_{2} & 0 & \left(2z-1)(xR_{2}+C_{3}-C_{5}\right) \end{array}\right) \end{array}$$ (16)

Based on Lyapunov’s theorem, the asymptotic stability of a system at each stability point can be determined by analyzing the eigenvalues of the Jacobi matrix [30]. Specifically, if all the eigenvalues of the Jacobi matrix have negative real parts, the equilibrium points are asymptotically stable; if the eigenvalues of the Jacobi matrix have one or more positive real parts, the points are unbalanced; if some of the eigenvalues of the Jacobi matrix are zero and others have negative real parts, the points are in a critical state and the stability needs to be further evaluated. The results of the stability analysis of each equilibrium point of the evolutionary game system are shown in Table 2.

**Table 2:** Analysis of system equilibrium points and their stability.

Equilibrium Points	Eigenvalue	Condition	Real Part Symbol	Stability
*E*_1_(0,0,0)	*R*_3_ + *F*,*U*_2_ + *C*_6_, *C*_5_ ‒ *C*_3_	**\**	+,+,×	unstable point
*E*_2_(1,0,0)	‒ *R*_3_ ‒ *F*,*U*_1_ + *C*_6_, *C*_5_ ‒ *R*_2_ ‒ *C*_3_	\	-,+,×	unstable point
*E*_3_(0,1,0)	*R*_3_ ‒ *π*_2_ + *F*, ‒*U*_2_ ‒ *C*_6_, *C*_5_ ‒ *C*_3_	1,3	-,-,-	ESS
*E*_4_(0,0,1)	*R*_3_ ‒ *π*_2_ + *F*,*U*_2_ ‒ *R*_4_ + *C*_6_,*C*_3_ ‒ *C*_5_	1,5,4	-,-,-	ESS
*E*_5_(1,1,0)	‒*R*_3_ ‒ *π*_2_ ‒ *F*, ‒*U*_1_ ‒ *C*_6_, ‒*R*_2_ ‒ *C*_3_ + *C*_5_	2,3	-,-,-	ESS
*E*_6_(1,0,1)	‒*R*_2_ ‒ *R*_3_ ‒ *F*,*U*_1_ ‒ *R*_4_ + *C*_6_, ‒ *R*_2_ ‒ *C*_3_ + *C*_5_	6,3	-,-,-	ESS
*E*_7_(0,1,1)	*π*_1_ + *R*_2_ + *R*_3_ ‒ *π*_2_ + *F*, ‒*U*_2_ + *R*_4_ ‒ *C*_6_, *C*_3_ ‒ *C*_5_	8	+,×,×	unstable point
*E*_8_(1,1,1)	‒*π*_1_ ‒ *R*_2_ ‒ *R*_3_ + *π*_2_ ‒ *F*, ‒*U*_1_ + *R*_4_ ‒ *C*_6_, *R*_2_ + *C*_3_ ‒ *C*_5_	8,7,9	-,-,-	ESS
*E*_9_(*x*_1_,1,*z*_1_)	*R*_4_*z* ‒ *Lc*_3_ ‒ *U*_2_ ‒ *U*_1_*x* + *U*_2_*x*, *λ*_2_, *λ*_3_	**\**	×,×,×	uncertain
*E*_10_(*x*_2_,*y*_2_,0)	*C*_5_ ‒ *C*_3_ ‒ *R*_2_*x*, *λ*_4_, *λ*_5_	**\**	×,×,×	uncertain
*E*_11_(*x*_3_,*y*_3_,1)	*C*_5_ ‒ *C*_3_ + 2(*C*_3_ ‒ *C*_5_)*z* ‒ *R*_2_*x* + 2*R*_2_*xz*, *λ*_6_, *λ*_7_	**\**	×,××,×	uncertain

Note: × indicates uncertain symbols; *x*_1_, *x*_2_, *x*_3_, *y*_2_, *y*_3_, *z*_1_ are all equilibrium point coordinates; *λ*_2_ ‒ *λ*_7_ are the eigenvalue corresponding to the equilibrium point. Condition 1: *R*_3_ + *F < π*_2_; condition 2: *R*_3_ + *F > π*_2_; condition 3: *C*_5_ < *C*_3_; condition 4: *C*_5_ > *C*_3_; condition 5: *U*_2_ + *C*_6_ < *R*_4_; condition 6: *U*_1_ + *C*_6_ < *R*_4_; condition 7: *U*_1_ + *C*_6_ > *R*_4_; Condition 8: *π*_1_ > *π*_2_; condition 9: *C*_5_ > *R*_2_ + *C*_3_.

Corollary 5: When *C*_5_ > *R*_2_ + *C*_3_ and *R*_4_ < *U*_1_ + *C*_6_, the replicated dynamical system has one and only one stable point *E*_8_(1,1,1). When *C*_5_ < *C*_3_, if *R*_3_ + *F* > *π*_2_ is satisfied, the replicated dynamical system has one and only one stable point *E*_5_(1,1,0); if *R*_4_ > *U*_1_ + *C*_6_ is satisfied, the replicated dynamical system has one and only one stable point *E*_6_(1,0,1).

Proof: When *C*_5_ > *R*_2_ + *C*_3_, i.e. condition 9 is satisfied, *E*_3_(0,1,0), *E*_5_(1,1,0) and *E*_6_(1,0,1) do not satisfy the stability condition, and if *R*_4_ < *U*_1_ + *C*_6_, i.e. condition 7 is satisfied, *E*_4_(0,0,1) is an unstable point, so the system has only one stable point *E*_8_(1,1,1). When *C*_5_ < *C*_3_, *R*_3_ + *F* > *π*_2_, namely conditions 2 and 3 are satisfied at the same time, *E*_5_(1,1,0) can be similarly obtained as the only stable point of the system. When *C*_5_ < *C*_3_ and *R*_4_ > *U*_1_ + *C*_6_, *E*_6_(1,0,1) is the only stable point in the system.

Corollary 5 shows differences in the choice of initial values(e.g. *C*_3_, *C*_5_, *R*_3_, *F*, etc.) can affect the strategy combination evolution of the parties in the three-party game system to stabilize at different stability points. For example, if the governments want to promote the healthy development of the new energy vehicle market by continuously reducing new energy subsidies, the three-party game system will tend to move from stable state *E*_8_(1,1,1) (i.e. car manufacturers produce new energy vehicles, consumers buy cars, and government departments increase subsidies) to state *E*_5_(1,1,0) (i.e. car manufacturers produce new energy vehicles, consumers buy cars, and government departments reduce or do not subsidize), The governments need to rationalize the cost of human resources spent on subsidy policy, control the time cost for society to shift from high-carbon to low-carbon green travel, increase the revenue earned by car companies through carbon trading management and appropriately increase the penalties for car companies that produce fuel cars.

## Simulation analysis and discussion

To further verify the stability of the three-way evolutionary game model, the parameters of the model are assigned specific different values in numerical simulations using MATLAB 2021a software. The initial values of the parameters in scenario 1 are set as follows: *x* = 0.27, *y* = 0.4, *z* = 0.64, *P*_1_ = 210000, *P*_2_ = 150000, *V*_1_ = 300000, *V*_2_ = 250000, *π*_1_ = 18000, *π*_2_ = 18000, *C*_1_ = 0.17. *C*_2_ = 0.66, *L* = 630, *C*_3_ = 500, *C*_4_ = 700, *C*_5_ = 60000, *C*_6_ = 240, *R*_1_ = 50000, *R*_2_ = 35000, *R*_3_ = 40000, *R*_4_ = 500, Λ = 15000, *n*_1_ = 0.3, *F* = 2000. Initial values of parameters for *x*, *y*, *z*, *P*_1_, *P*_2_, *V*_1_, *V*_2_, *π*_1_, *π*_2_ refer to literatures [4, 5, 7], and other parameters refer to literatures [5, 10, 16, 26]. According to Corollary 5, when *C*_5_ > *R*_2_ + *C*_3_ and *R*_4_ < *U*_1_ + *C*_6_, the replicated dynamic system has one and only one stability point *E*_8_(1,1,1). This means in the early stages of the development of new energy vehicles, the probability of car producers producing new energy vehicles increases with the probability of government subsidies for new energy vehicles and the probability of consumers purchasing cars, eventually reaching a combination of evolutionary stability strategies. This is consistent with what are described in the literature [15]. On this basis, the impacts of *R*_2_, *R*_3_, *F*, *P*_1_, *V*_1_, *n*_1_, *C*_5_, *C*_3_ and *R*_4_ on the process of the evolutionary game are analyzed respectively.

**Fig 5:**
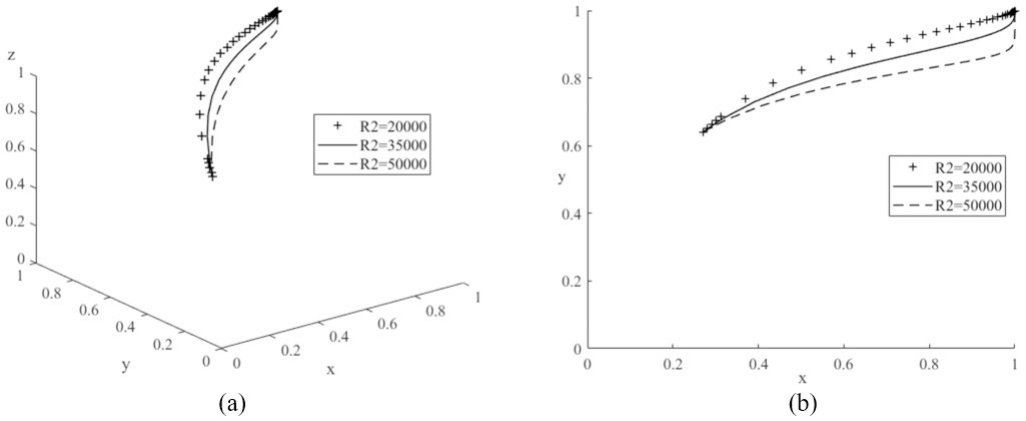
Impacts of different government subsidies (*R*_2_) on evolutionary game system.

**Fig 6:**
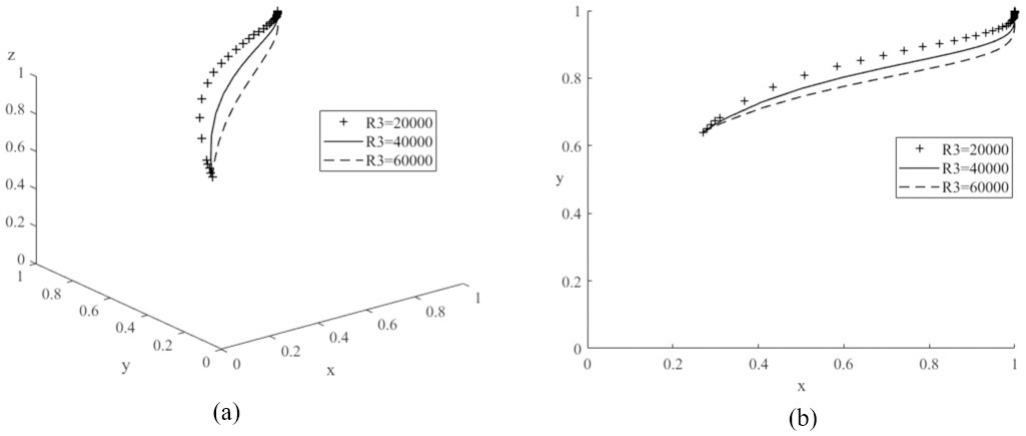
Impacts of different additional benefits (*R*_3_) through carbon trading on evolutionary game system.

**Fig 7:**
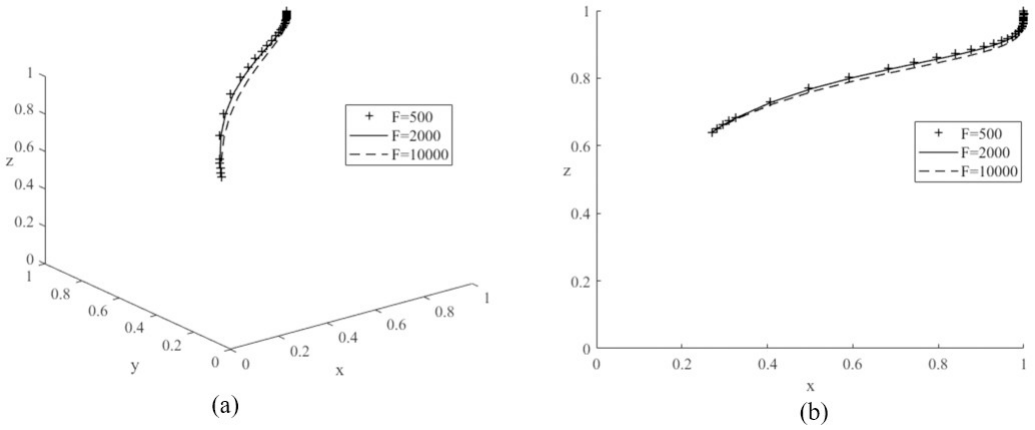
Impacts of different government fines (*F*) on evolutionary game system.

First, to analyze the effect of changing the value of *R*_2_ on the evolutionary game system process, the values of *R*_2_ are set to 20,000 (low value), 35,000 (medium value) and 50,000 (high value) respectively, and the average simulation results obtained by simulating the model 50 times are shown in Fig 5. The graph shows that the probability of the automobile manufacturer producing new energy vehicles increases with the probability of government subsidies for new energy as the system gradually converges to a stable point, and it is the same as literature [26]. In addition, the probability of the auto manufacturer producing new energy vehicles increases as the value of *R*_2_ continues to increase. Therefore, this result demonstrates the increase in government subsidies received by car manufacturers for new energy vehicles will provide a significant incentive for car manufacturers to produce more new energy vehicles [13]. Similarly, values of *R*_3_ are set to 20,000 (low value), 40,000 (medium value) and 60,000 (high value) respectively and the model is simulated 50 times as shown in Fig 6. The graph shows that an increase in the value of *R*_3_ will also make car manufacturers inclined to produce more new energy vehicles, so the governments should improve the local carbon emissions trading market [24], so that more vehicle manufacturers can gain more revenue through carbon trading. Then, *F* are assigned to 500 (low value), 2000 (medium value) and 10000 (high value) respectively, and the simulation results are shown in Fig 7. It can be seen an increase in the value of *F* will increase the cost of car manufacturers, reduce the production of traditional fuel cars, and produce more new energy cars. Therefore, to increase the production of new energy vehicles, the governments can also take some other indirect measures, such as appropriately increasing the penalties for traditional fuel vehicles [18]. The above analyses also confirm the results of Corollary 2, i.e., increasing the government subsidies received by car companies, the additional benefits gained by car companies from the management of the carbon trading scheme and the fines imposed on car companies producing conventional fuel vehicles will encourage car companies to produce more new energy vehicles.

**Fig 8:**
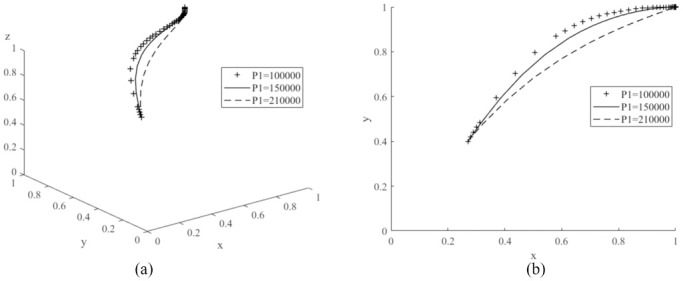
Impacts of various price (*P*_1_) of new energy vehicles on evolutionary game system.

**Fig 9:**
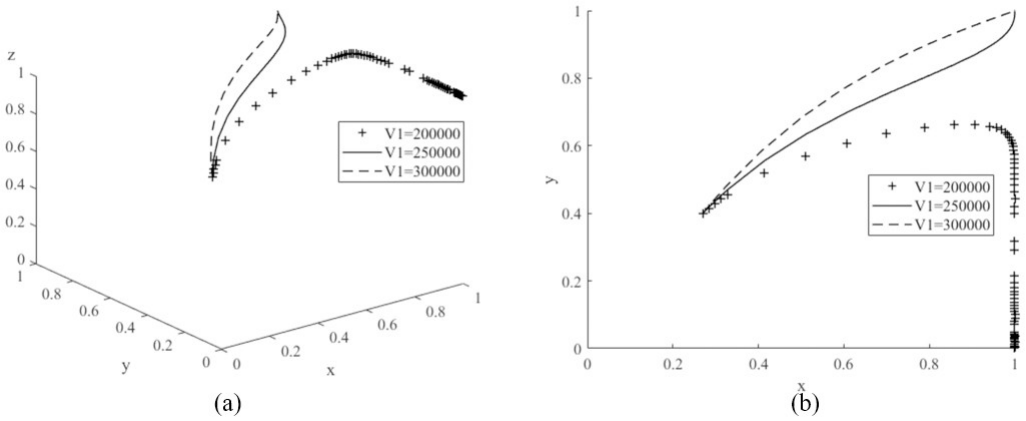
Impacts of various consumer utility value (*V*_1_) of new energy vehicles on evolutionary game system.

**Fig 10:**
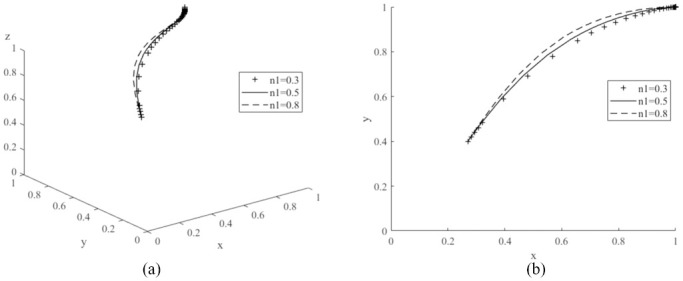
Impacts of various coverage of public charging posts (*n*_1_) on evolutionary game system.

The values of *P*_1_ are assigned to 100,000 (low value), 1500,000 (medium value) and 210,000 (high value) respectively, and the average simulation results obtained by evolving the replication dynamic equation 50 times are shown in Fig 8. From the graph, the probability of consumers purchasing new energy vehicles decreases significantly as the value of *P*_1_ continues to increase. Therefore, the governments need to focus on the sales price of new energy vehicles when promoting the new energy market. New energy vehicles are more competitive with a lower price [17]. Then, *V*_1_ is assigned to 200,000 (low value), 250,000 (medium value) and 300,000 (high value) respectively, and the simulation results are shown in Fig 9. The probability of purchasing new energy vehicles increases as the value of *V*_1_ increases. Compared to literature [22], it is worth noting that if the value of *V*_1_ is too low, it may shift the evolutionary game system to another stable point *E*_6_(1,0,1), where the probability of consumers purchasing cars decreases and eventually reaches zero, demonstrating that a change in the value of *V*_1_ can have an important impact on the game system. Let *n*_1_ be values of 0.3 (low), 0.5 (medium) and 0.8 (high) respectively, and the simulation results are shown in Fig 10. As the value of *n*_1_ increases, the probability of consumer purchasing new energy vehicles increases.

Therefore, for consumers to increase their purchases of new energy vehicles, manufacturers need to lower the price of new energy vehicles [17], have differentiated core technologies, improved the brand appeal of their products, and increased the utility value of their products in the minds of consumers. Governments also need to increase the coverage of charging piles in their cities. The above analyses verify the conclusion of Corollary 3, that the probability of car consumers purchasing new energy vehicles is inversely proportional to the sales price of new energy vehicles and positively proportional to the utility value of new energy vehicles to consumers and the coverage of charging piles in the city where they are located.

**Fig 11:**
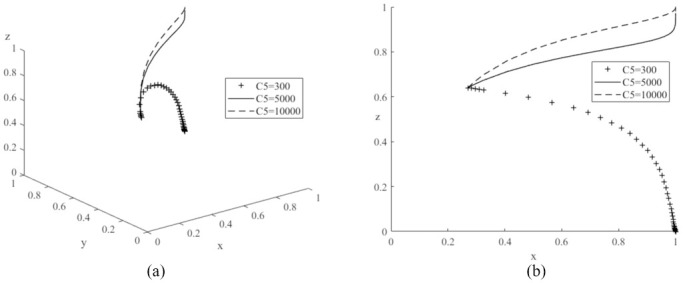
Impacts of various time cost (*C*_5_) of society’s shifting from high-carbon to low-carbon travels on evolutionary game system.

**Fig 12:**
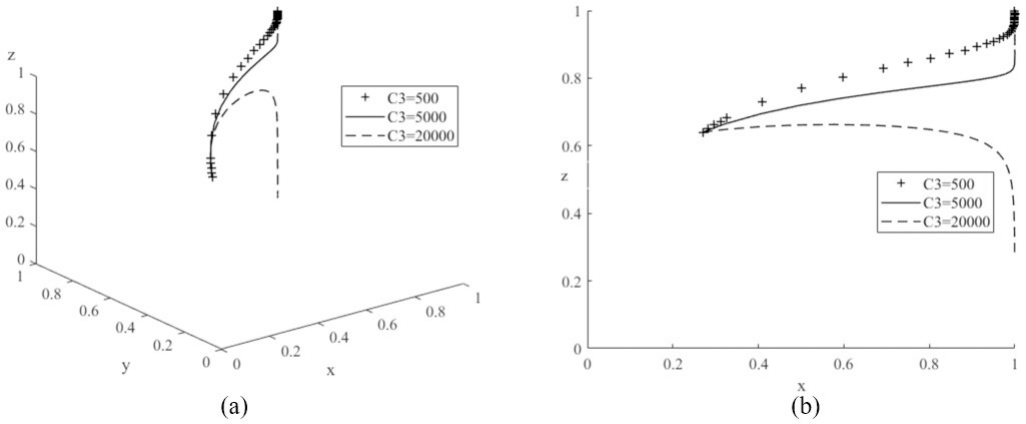
Impacts of various human resource costs (*C*_3_) of governments with subsidy policy on evolutionary game system.

Next, let *C*_5_ be values of 300 (low), 5,000 (medium) and 10,000 (high) respectively, and the simulation results are shown in Fig 11. As the value of *C*_5_ increases, the probability of government subsidies for new energy policies rises. Compared to literature [22], it is also worth noting that if the value of *C*_5_ is too low, it can shift the evolutionary game system to another stable point *E*_5_(1,1,0) where the probability of government subsidies for new energy vehicles decreases and tends to zero, demonstrating that a change in the value of *C*_5_ can have an important impact on the game system. Let *C*_5_ be 500 (low), 5,000 (medium) and 10,000 (high) respectively, and the simulation results are shown in Fig 12. As the value of *C*_5_ continues to increase, the probability of government subsidies for new energy policies decreases significantly. Combined with the results in Fig 5, the probability of government subsidies for new energy policies decreases as the value of *R*_2_ increases. The above simulation results verify Corollary 4, which states that the probability of government subsidies for new energy policies is positively proportional to the time cost for society to switch from high-carbon and high-pollution travel to low-carbon and green travel, and inversely proportional to the human resource costs of the governments with subsidized policies and the subsidy expenditure received by the production of new energy vehicles.

**Fig 13:**
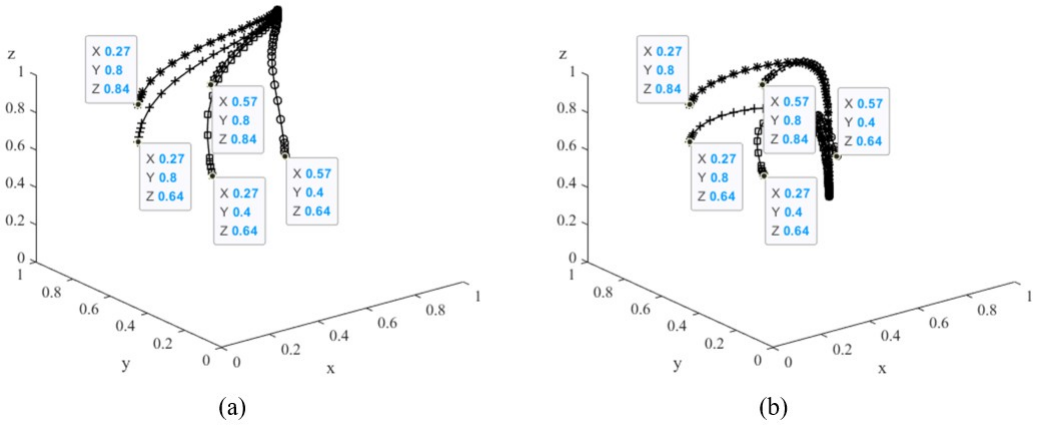
Simulation results of the evolution system in scenario 1 and 2 with different initial combination strategies.

The initial values of the parameters in scenario 2 are basically the same as those in scenario 1, except for *C*_3_ = 5000, *C*_5_ = 300, *R*_3_ = 60000 and *F* = 10000. According to the conclusions of Corollary 5, scenario 2 satisfies the following conditions, namely *C*_5_ < *C*_3_, *R*_3_ + *F* > *π*_2_. It means there should be only one stable point *E*_5_(1,1,0) in the evolutionary system, i.e., in the late stage of the development of new energy vehicles, car manufacturers produce new energy vehicles, consumers buy them, and the governments don’t take subsidies. The two scenarios were evolved 50 times from different initial strategy combinations. The initial strategy combinations are shown in Table 3, the simulation results obtained are shown in Fig 13, and the results in the figure are consistent with the conclusions of Corollary 5.

**Table 3:** Initial strategy combinations for scenario 1 and scenario 2.

Initial Strategy Combination	Car Manufacturers *x*	Consumers *y*	Governments *z*
1	0.27	0.4	0.64
2	0.27	0.8	0.64
3	0.27	0.8	0.84
4	0.57	0.4	0.64
5	0.57	0.8	0.84

As can be seen from Fig 13, in the later stages of the development of new energy vehicles (i.e. in 2060), when the time cost (*C*_5_) for society to gradually shift from high-carbon to low-carbon travel is low, or even lower than the human cost (*C*_3_), the governments tend to reduce subsidies in order to achieve the “double carbon” target. On the other hand, the governments also tend to reduce direct subsidies if a set of indirect policies, such as additional benefits for car companies through carbon trading management (*R*_3_) and government penalties for conventional car production (*F*), can achieve better results. This suggests that to achieve the “double carbon” target, the governments should consider a combination of direct and indirect policies to promote the rapid development of the new energy vehicle industry as soon as possible. If the governments intend to gradually reduce or even eliminate direct policy subsidies in the future due to financial pressures, it is crucial that indirect policies should play a critical role.

## Conclusions and recommendations

To attain the objectives of reaching the "carbon peak" by 2030 and "carbon neutrality" by 2060, it is imperative for governments to take an active approach in advocating for environmentally friendly modes of transportation, such as low-carbon and green transportation. This study focuses on new energy vehicles in the context of "double carbon" and examines the game strategies of various stakeholders, including vehicle manufacturers, consumers, and government agencies, as well as the equilibrium conditions of the evolutionary game system. By constructing a three-party evolutionary game model, the paper presents relevant measures and suggestions based on the results of the simulations to support the validity of the findings.

The main conclusions are as follows:(1) There is a strong correlation between government subsidies, production, and sales of new energy vehicles. In the early stages of new energy vehicle development, government subsidies as an evolutionary stabilization strategy are still important for the rapid growth of new energy vehicles production and sales, which is verified by Corollary 1. As the new energy vehicle industry matures, indirect policies can play a key role in regulating the new energy vehicle market, which is also confirmed by Corollary 2. (2) The price of new energy vehicles is an important influencing factor because consumers are sensitive to the price of new energy vehicles. In addition, the attractiveness of vehicle brands, the perceived utility of the products among consumers, and the coverage of charging infrastructure in cities also determine whether consumers choose to purchase and use new energy vehicles, which is shown in Corollary 3. (3) If the government chooses to adopt a direct subsidy policy, it needs to focus on factors such as the time cost of switching from high-carbon travel to low-carbon travel and the human cost of the government’s subsidy policy, which are suggested by Corollary 4. If the government opts for an indirect subsidy policy, it needs to increase the revenues generated by car companies through the administration of the Emission Trading Scheme [19] and increase the penalties for car companies that produce fuel cars accordingly, as shown in Corollary 5.

The practical implications are given:(1) The government needs to be flexible in its use of direct subsidies and indirect policies and carefully consider the impact that different factors can have on the effectiveness of policy implementation. (2) Car manufacturers need to adopt different sales policies for different groups of consumers. For example, for price-sensitive consumers, the price of new energy vehicles should be reduced as much as possible to encourage purchases [17], but for other consumers, the attractiveness of vehicle brands and the perceived utility of the products among consumers may be more important. (3) In addition to encouraging consumers to buy new energy vehicles, the government also needs to consider how convenient it is for consumers to use them. Providing the number of charging stations in the city, rationally planning the coverage, and completing the unified management of different brands of charging piles are equally important to encourage people to use new energy vehicles as their top mode of daily transportation.

There are still some shortcomings in this paper that need to be addressed. In particular, the paper only examines the evolutionary game dynamics between vehicle manufacturers, users, and governments and dose not consider the influence of news media reports, whether positive or negative, on the sales and production of new energy vehicles. In addition, the parameter settings in the paper do not fully reflect the real situation and the results of the numerical simulations may be biased. Therefore, incorporating additional variables such as news media and improving the precision and accuracy of the simulation results are important research directions for future studies.

## Supporting information

S1 Dataset (ZIP)

## References

[1] China Government.com. Notice of the State Council on Printing and Distributing: The Action Plan for Carbon Peaking by 2030 [cited 2022 June 1]. http://gxt.hebei.gov.cn/hbgyhxxht/xwzx32/snxw40/889348/index.html.

[2] Phoenix.com. Under the "3060 Double Carbon" Goal, the Challenges and Opportunities Faced by Carbon Reduction in the Automobile Industry[cited 2021 November 1]. https://auto.ifeng.com/quanmeiti/20211101/1658971.shtml.

[3] Xinhua News Agency. Opinions of the Central Committee of the Communist Party of China and the State Council on Completely, Accurately and Comprehensively Implementing the New Development Concept and Doing a Good Job in Carbon Neutrality[cited 2021 October 24]. http://www.gov.cn/zhengce/2021-10/24/content_5644613.htm.

[4] Gao Q, Fan M, Du JG. Evolutionary study of the impact of government subsidies on new energy vehicle enterprises. Science and Technology Management Research.2014;34:5–79.

[5] Li W, Long R, Chen H. Consumers’ evaluation of national new energy vehicle policy in China: An analysis based on a four-paradigm model. Energy Policy.2016;99:33–41.

[6] China Economic Times. The Demand for New Energy Vehicles Will Be Released under The "Dual Carbon" Goal[cited 2021 August 30]. https://xw.qq.com/cmsid/20210830A047T200?pgv_ref=baidutw.

[7] Huck S, Oechssler J. The indirect evolutionary approach to explaining fair allocations. Games and Economic Behavior. 1999;28:13–24.

[8] Qin S, Xiong Y. Innovation strategies of Chinese new energy vehicle enterprises under the influence of non-financial policies: Effects, mechanisms and implications. Energy Policy. 2022; 164, 112946.

[9] Liao H, Peng S, Li L. The role of governmental policy in game between traditional fuel and new energy vehicles. Computers & Industrial Engineering. 2022;108292.

[10] Han J, Guo JE, Cai X. An analysis on strategy evolution of research & development in cooperative innovation network of new energy vehicle within policy transition period. Omega. 2022;102686.

[11] Pan Y, Dong F. Dynamic evolution and driving factors of new energy development: fresh evidence from China. Technological Forecasting and Social Change.2022; 176:121475.

[12] Trosta T, Sterner M, Bruckner T. Impact of electric vehicles and synthetic gaseous fuels on final energy consumption and carbon dioxide emissions in Germany based on long-term vehicle fleet modelling. Energy.2017;141:5–25.

[13] Lin B, Shi L. Do environmental quality and policy changes affect the evolution of consumers’ intentions to buy new energy vehicles. Applied Energy. 2022; 310:118582.

[14] Gong H, Wang MQ, Wang H. New energy vehicles in China: policies, demonstration, and progress. Mitigation and Adaptation Strategies for Global Change. 2013; 18:207–228.

[15] Zhong TY, Du R. Research on new energy vehicle subsidy strategy based on game theory. China Management Science. 2015; 23:817–822.

[16] Zhang L, Song Y, Zhang M, Wu WQ. Evolutionary game analysis of strategic interaction of environmental regulation among local governments. Environmental Development. 2023; 45: 100793.

[17] Zhou Y, Pan Y. Financial subsidies and tax breaks-An analysis of new energy vehicle industrial policy from the perspective of transaction costs. Management World. 2019; 35:133–149.

[18] Zhang XL, Wang JJ. Analysis of government subsidies in the supply chain of new energy vehicles based on Shapley’s value method. Soft Science.2015;29:54–58.

[19] Zhang SY, Wang CX, Yu C. The evolutionary game analysis and simulation with system dynamics of manufacturer’s emissions abatement behavior under cap-and-trade regulation. Applied Mathematics and Computation. 2019; 355:343–355.

[20] Ma SH, Tan H, Dai YS. Analysis of consumer characteristics and preferences in the new energy vehicle market. Industrial Technology and Economics. 2013; 32:13–121.

[21] Xu GH, Xu F. Study on the factors influencing the purchase decision of new energy vehicles. China Population, Resources and Environment. 2010; 20:91–95.

[22] Chen K, Gu R, Hu J. A study on perceived benefit-perceived risk framework: A study on the purchase intention of new energy vehicles based on the perceived benefit-perceived risk framework. Journal of Nanjing University of Technology. 2019; 18:61–70.

[23] Helveston JP, Liu Y, Feit MD, Fuchs E, Klampfl E, Michalek JJ. Will subsidies drive electric vehicle adoption? Measuring consumer preferences in the U.S. and China. Transportation Research Part A-Policy and Practice. 2015; 73:96–112.

[24] Li XW, Huang RN, Dai JC, Li JR, Shen Q. Research on the evolutionary game of construction and demolition waste (CDW) recycling units’ green behavior, considering remanufacturing capability. International Journal of Environmental Research and Public Health. 2021; 18(17): 9268. doi: 10.3390/ijerph18179268 34501858 PMC8431377

[25] You Q, Yu K, Zhou LJ, Zhang J, Lv MY, Wang JS. Research on risk analysis and prevention policy of coal mine workers’ group behavior based on evolutionary game. Resources Policy. 2023; 80: 103262.

[26] Ji SF, Zhao D, Luo RJ. Evolutionary game analysis on local governments and manufacturers’ behavioral strategies: Impact of phasing out subsidies for new energy vehicles. Energy.2019; 189:1–16.

[27] Shi YY, Wei ZX, Shahbaz M, Zeng YC. Exploring the dynamics of low-carbon technology diffusion among enterprises: An evolutionary game model on a two-level heterogeneous social network. Energy Economics. 2021;101: 105399.

[28] Syed ARK, Zhang Y, Khalid F. Green capabilities, green purchasing, and triple bottom line performance: Leading toward environmental sustainability. Business Strategy and the Environment.2022.

[29] Khan SAR, Yu Z, Umar M, Zia-ul-haq, HM, Tanveer M, Janjua LR. Renewable energy and advanced logistical infrastructure: Carbon‐free economic development. Sustainable Development. 2022; 30(4): 693–702.

[30] Dou YD, Sun XL, Ji AK, Wang YN, Xue XL. Development strategy for prefabricated construction projects: A tripartite evolutionary game based on prospect theory. Engineering. Construction and Architectural Management. 2023; 30(1): 105–124.

[31] Wang G, Chao YC, Jiang TL, Lin JQ, Peng HC, Chen HT, et al. Analyzing the effects of government policy and solar photovoltaic hydrogen production on promoting CO2 capture and utilization by using evolutionary game analysis. Energy Strategy Reviews. 2023; 45: 101044.

[32] Zhou CY, He JR, Li YJ, Chen WL, Zhang Y, Zhang H, et al. Green Independent Innovation or Green Imitation Innovation? Supply Chain Decision-Making in the Operation Stage of Construction and Demolition Waste Recycling Public-Private Partnership Projects. Systems. 2023; 11(2): 94.

[33] Jiang N, Feng YQ, Wang XJ. Fractional-order evolutionary game of green and low-carbon innovation in manufacturing enterprises. Alexandria Engineering Journal. 2022;61(2): 12673–12687.

[34] Zhang X, Bai X. Incentive policies from 2006 to 2016 and new energy vehicle adoption in 2010–2020 in China. Renewable & Sustainable Energy Reviews. 2017; 70:24–43.

[35] Hawkins TR, Singh B, Majeau BG. Comparative environmental life cycle assessment of conventional and electric vehicles. Journal of Industrial Ecology. 2012; 17:53–64.

[36] Yuan X, Liu X, Zuo J. The development of new energy vehicles for a sustainable future: a review. Renewable & Sustainable Energy Reviews. 2015; 42:298–305.

[37] Zhao J, Rao Z, Huo Y. Thermal management of cylindrical power battery module for extending the life of new energy electric vehicles. Applied Thermal Engineering. 2015; 85:33–43.

