# Supplementary figures and images for: Exploring the interplay of new energy vehicle enterprises, consumers, and government in the context of the "dual carbon" target: An evolutionary game and simulation analysis
Source: PLoS One. 2023 Sep 8;18(9):e0291175. doi: 10.1371/journal.pone.0291175 (PMC10490975; doi:10.1371/journal.pone.0291175)

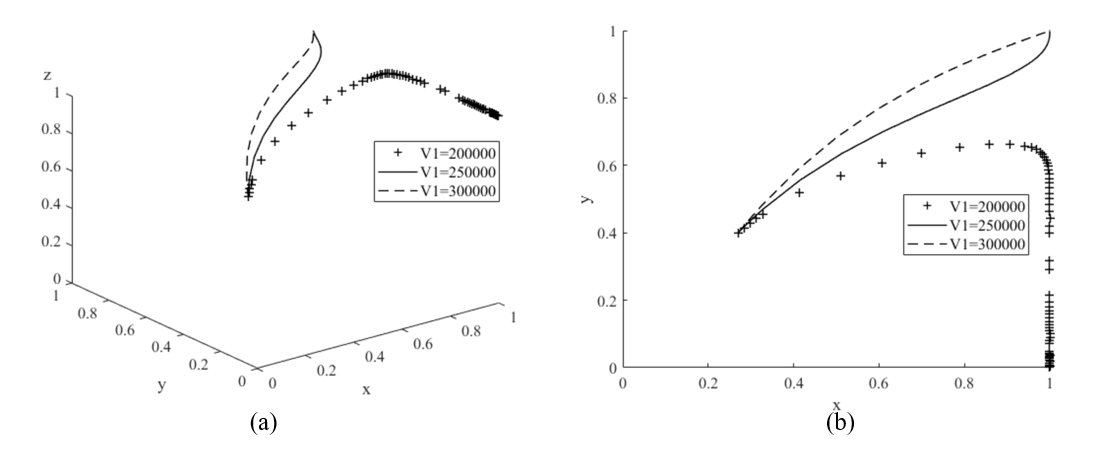

Supplement: S1 Dataset — (ZIP) [file pone.0291175.s001.zip › minimal data set/figures/Fig 9.tif]

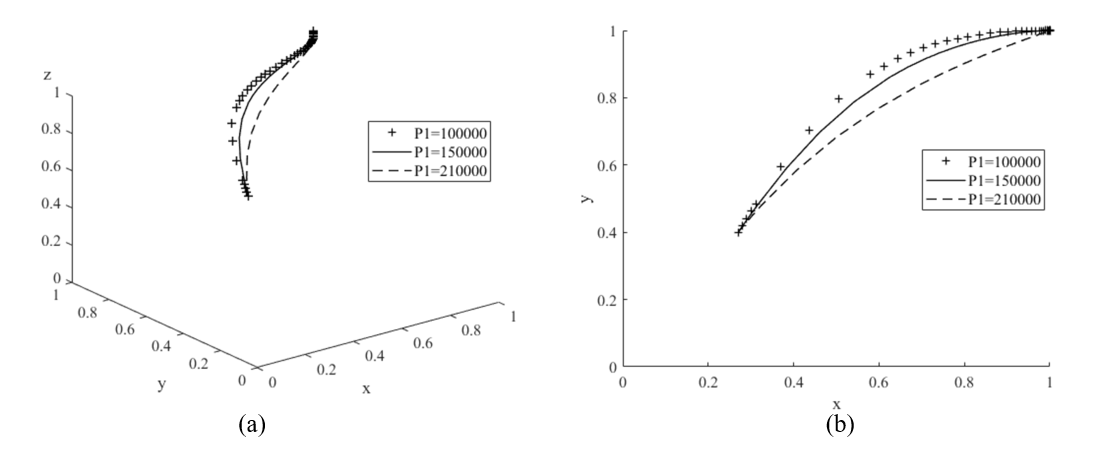

Supplement: S1 Dataset — (ZIP) [file pone.0291175.s001.zip › minimal data set/figures/Fig 8.tif]

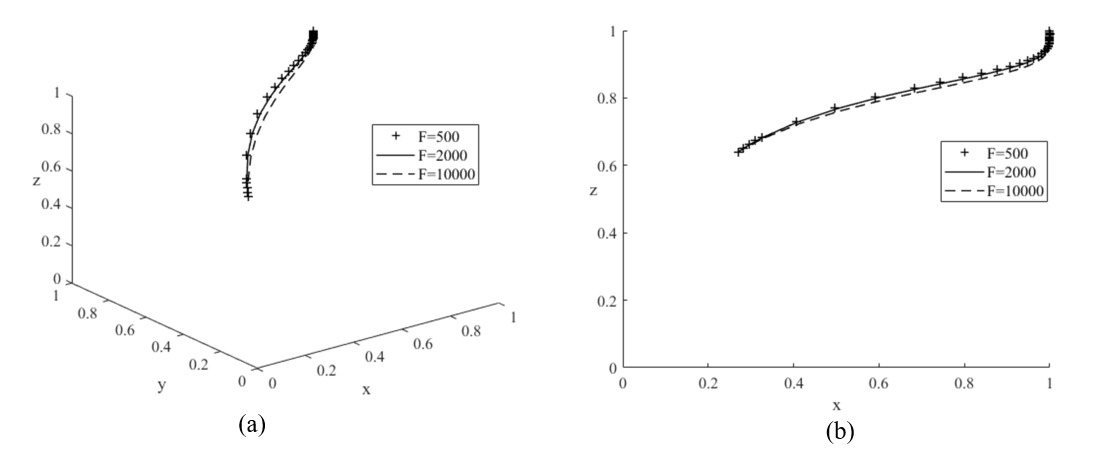

Supplement: S1 Dataset — (ZIP) [file pone.0291175.s001.zip › minimal data set/figures/Fig 7.tif]

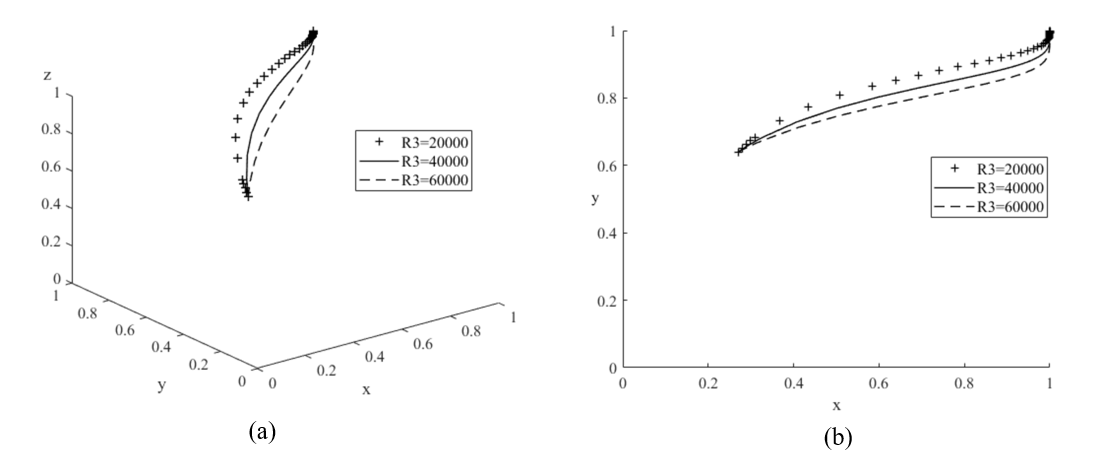

Supplement: S1 Dataset — (ZIP) [file pone.0291175.s001.zip › minimal data set/figures/Fig 6.tif]

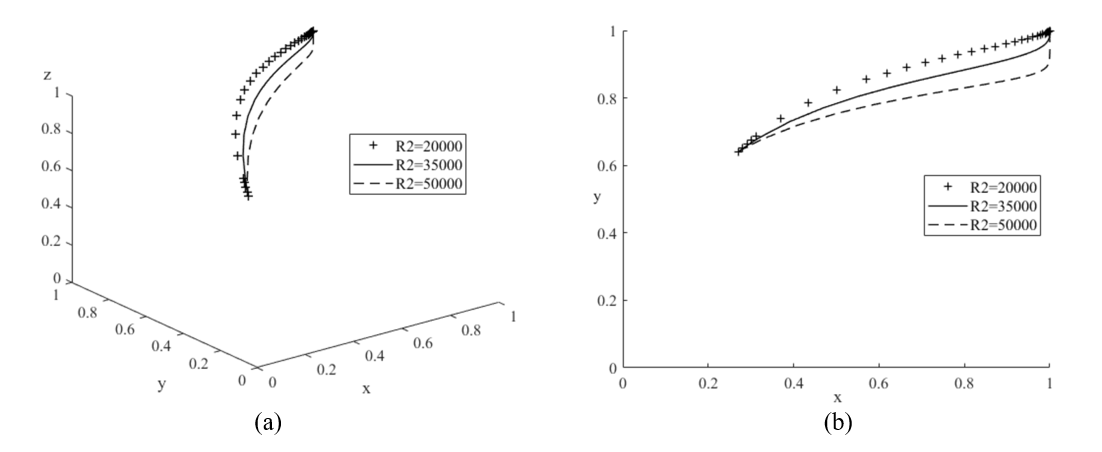

Supplement: S1 Dataset — (ZIP) [file pone.0291175.s001.zip › minimal data set/figures/Fig 5.tif]

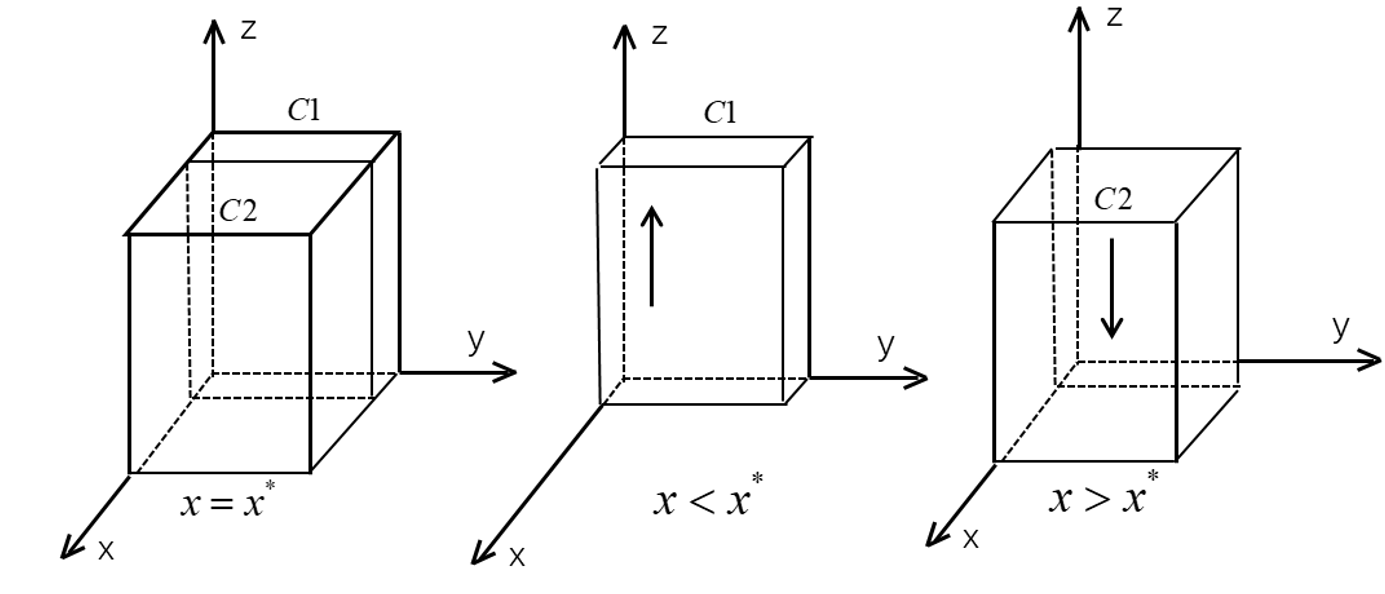

Supplement: S1 Dataset — (ZIP) [file pone.0291175.s001.zip › minimal data set/figures/Fig 4.tif]

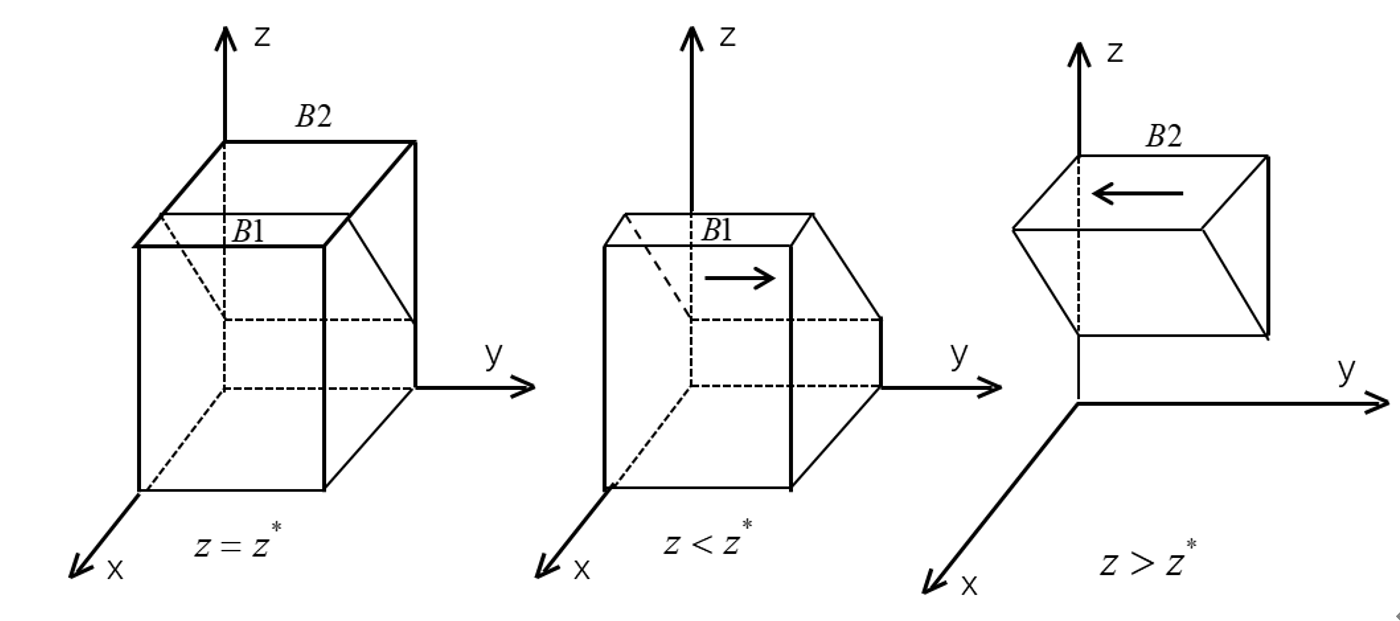

Supplement: S1 Dataset — (ZIP) [file pone.0291175.s001.zip › minimal data set/figures/Fig 3.tif]

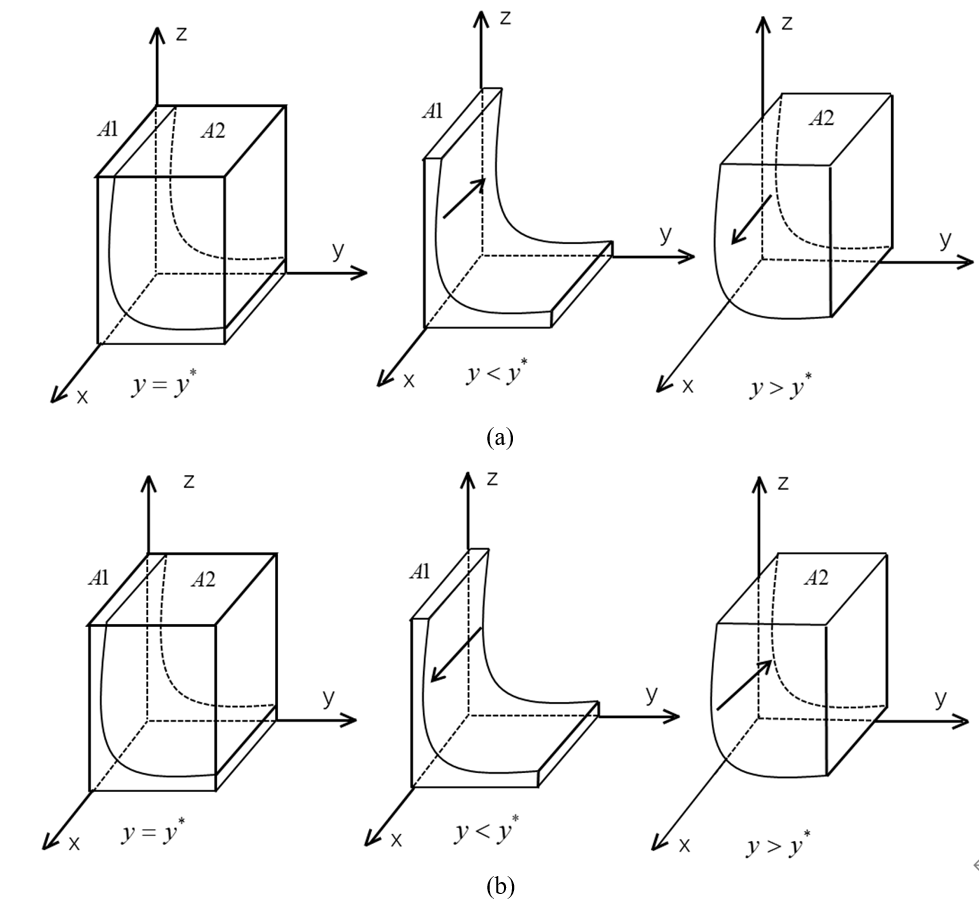

Supplement: S1 Dataset — (ZIP) [file pone.0291175.s001.zip › minimal data set/figures/Fig 2.tif]

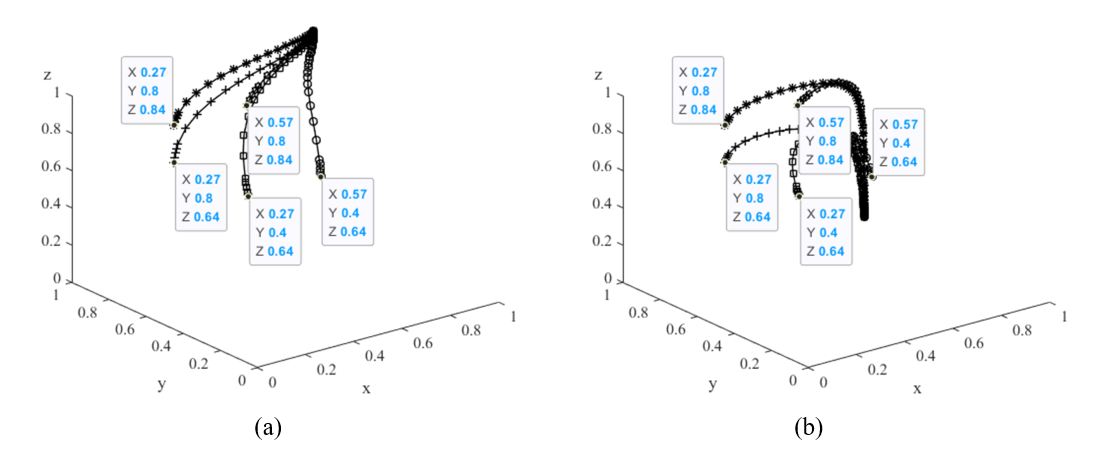

Supplement: S1 Dataset — (ZIP) [file pone.0291175.s001.zip › minimal data set/figures/Fig 13.tif]

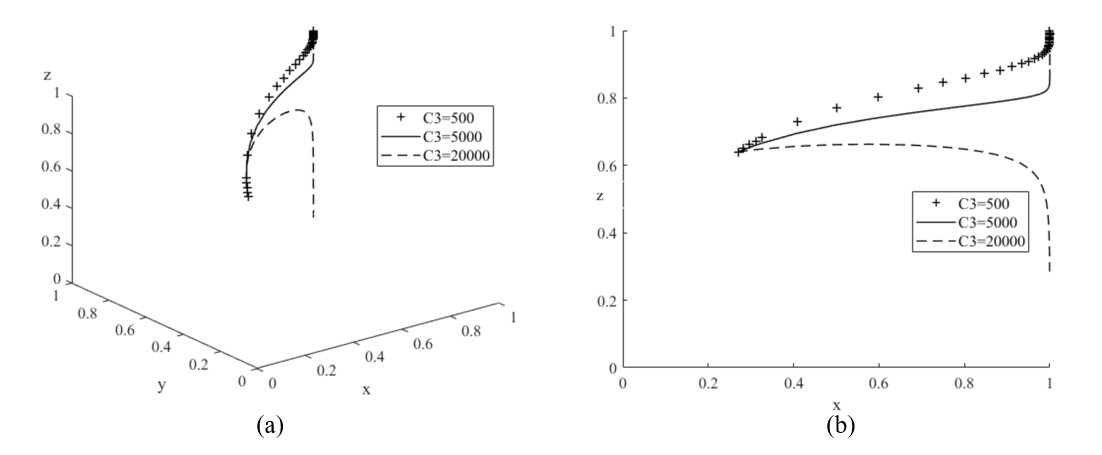

Supplement: S1 Dataset — (ZIP) [file pone.0291175.s001.zip › minimal data set/figures/Fig 12.tif]

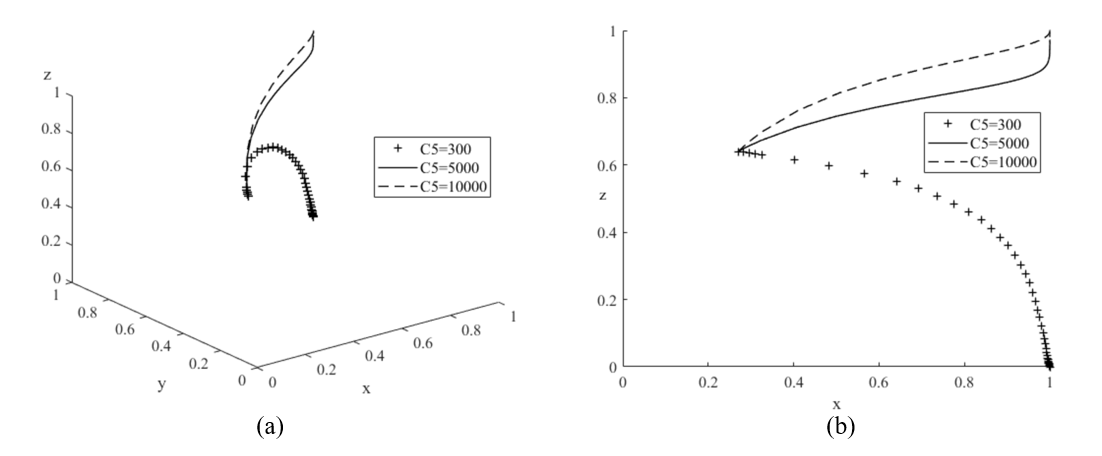

Supplement: S1 Dataset — (ZIP) [file pone.0291175.s001.zip › minimal data set/figures/Fig 11.tif]

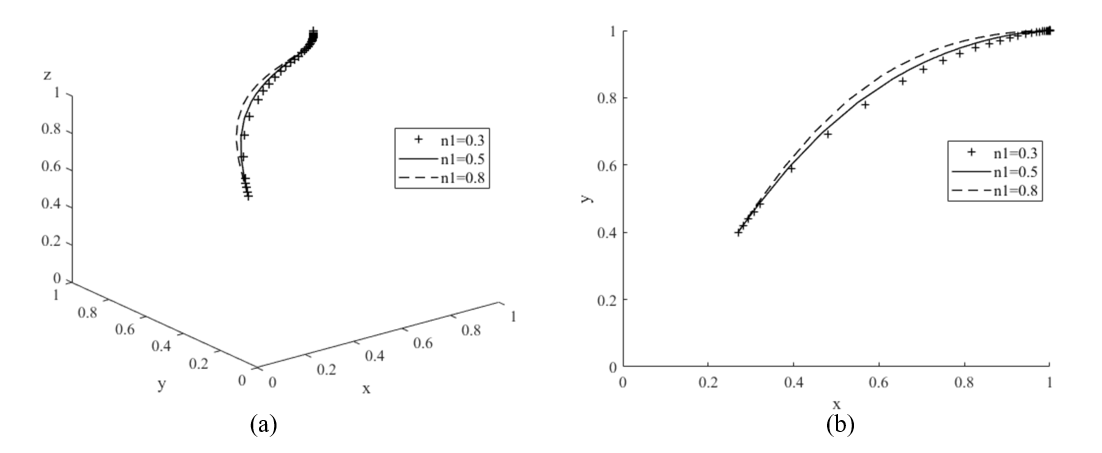

Supplement: S1 Dataset — (ZIP) [file pone.0291175.s001.zip › minimal data set/figures/Fig 10.tif]

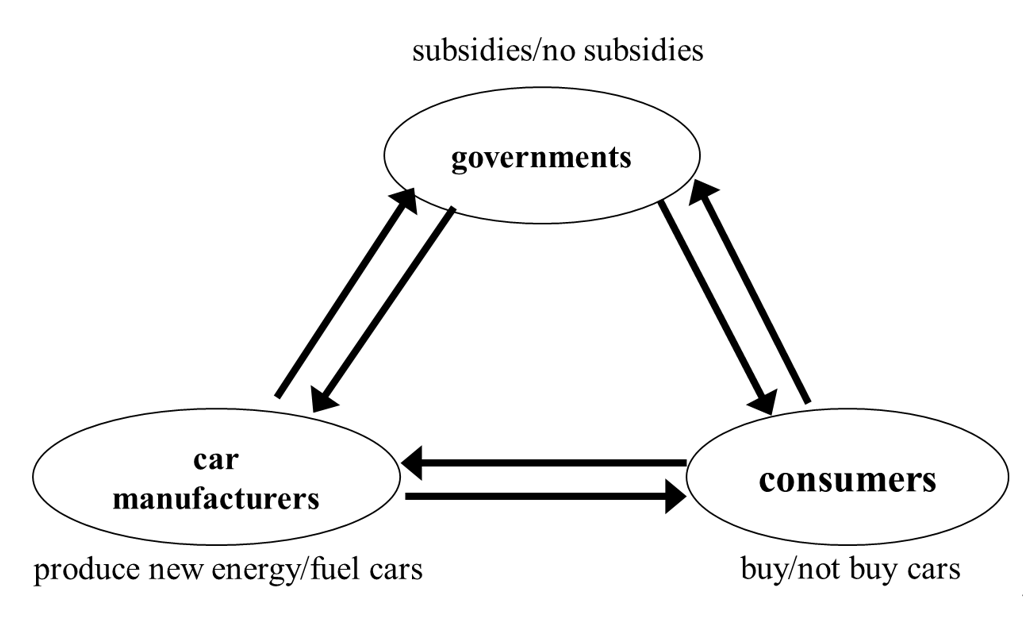

Supplement: S1 Dataset — (ZIP) [file pone.0291175.s001.zip › minimal data set/figures/Fig 1.tif]
